# Zn^2+^ detection of a benzimidazole 8-aminoquinoline fluorescent sensor by inhibited tautomerization†

**DOI:** 10.1039/d1ra05591g

**Published:** 2021-11-11

**Authors:** Harun Taş, Jörg Adams, Jan C. Namyslo, Andreas Schmidt

**Affiliations:** Clausthal University of Technology, Institute of Organic Chemistry Leibnizstrasse 6 D-38678 Clausthal-Zellerfeld Germany schmidt@ioc.tu-clausthal.de; Clausthal University of Technology, Institute of Physical Chemistry Arnold-Sommerfeld-Strasse 4 D-38678 Clausthal-Zellerfeld Germany

## Abstract

A new fluorescent chemosensor based on 8-aminoquinoline L1 bearing a benzimidazole moiety was synthesized, which exists as two predominant tautomers L1A and L1B in diluted DMSO-d_6_ solution. Among various metal ions, L1 showed a highly selective and sensitive turn-on fluorescence response to the presence of Zn^2+^ ions in methanol. The detection limit for Zn^2+^ by L1 was calculated to be 1.76 × 10^−7^ M. The 1 : 1 complexation ratio of the L1–Zn complex was confirmed through Job plot measurements. Complexation studies were performed by FT-IR, NMR and HR-ESI MS measurements and DFT calculations. With the gained insight, it was possible to successfully apply L1 in water sample analysis.

## Abstract 2

The sensor shows a high selectivity and sensitivity toward zinc cations, accompanied by a distinct green fluorescence emission.

## Graphical abstract

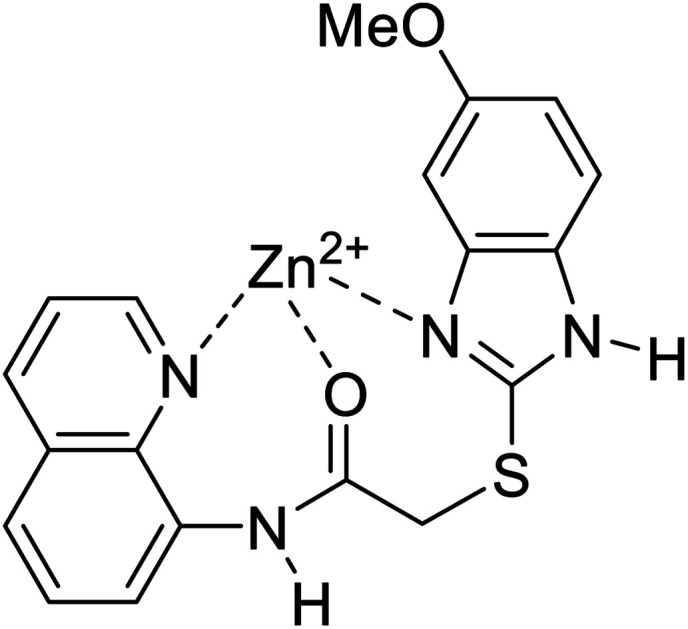

## Introduction

Zinc is known to be a common essential element to all living organisms. It has proven to be of vital importance to various biological processes such as enzyme activity or DNA protection.^1^ Though mostly present in complexed form,^2^ a lack or excess of zinc causes disturbances in biological systems and is often linked to a series of diseases, *e.g.* epilepsy, Alzheimer's disease or hypoxia ischemia.^3^ Apart from biological processes, excess zinc concentrations cause environmental problems. Thus, soil microbial activities are unfavorably affected which subsequently leads to phytotoxicity and, in general, to lowered crop quality.^4^ This is particularly problematic in former mining regions such as the Harz mountains (Lower Saxony, Germany) where we are located. Increased zinc concentrations are not only caused by contaminated sites as a result of centuries of mining activity, but also unintentionally by the local metal industry.^5^

Zinc is difficult to detect spectroscopically due to its 3d^10^ electron configuration. Even though various methods are known for its detection, most of them have not proven to be field-operational. Oftentimes, expensive equipment and extensive sample preparations are necessary for obtaining results.^6^ Therefore, the demand for low cost and easy monitoring systems has been high. Fluorescent chemosensors have proven to be a valuable asset in the sensing and monitoring of heavy and transition metal ions.

Numerous fluorescent chemosensors for Zn^2+^-sensing have been designed over the past decades.^7,8^ Among many trace metal ion sensors, quinoline is a common fluorophore used as a backbone in zinc sensing structures.^9,10^ The application of various 8-hydroxyquinolates in the fluorometric detection of zinc dates back as far as the 1960s.^11^ 8-Aminoquinoline based Zn^2+^-chemosensors are also known.^10,12,13^ However, fluorogenic Zn^2+^-sensors are not limited to quinoline structures only. Contemporary examples range from systems based on *e.g.* coumarin,^14^ fluorescein,^15^ benzimidazole^16,17^ or silsesquioxane.^18^ Further contemporary examples can be found in Table S1 (ESI†).

Unfortunately, the interference of same group metals such as Hg or Cd can cause severe problems in Zn^2+^ detection due to their similar properties.^19,20^ Therefore, there is a great interest in the design of chemosensors that are easily synthesized, have a high sensitivity, a high response and can discriminate between Zn^2+^ and Cd^2+^/Hg^2+^ in real time.

The implementation of additional heteroatom containing fragments have been reported to enhance chelation abilities.^21^ Intramolecular hydrogen bonds are often observed between binding units present in aminoquinoline chemosensors. Upon addition of metal ions, these hydrogen bonds can be broken by chelation resulting in a fluorescence emission due to stronger ICT processes.^12,22^ On the other hand, it has been reported that the inhibition of prototropic tautomerization phenomena in benzimidazole fragments can be accompanied by a fluorescence response.^16,17^

In continuation of our interest in metal adducts and metal complexes of heterocycles such as mesomeric betaines and *N*-heterocyclic carbenes^23^ in catalysis,^24^ metal recovery and recycling,^25^ we report here on a new 8-aminoquinoline based chemosensor L1 for the Zn^2+^ detection. L1, bearing a benzimidazole moiety, was acquired through a simple two-step synthesis and exhibits a prototropic tautomerization, which was spectroscopically proven to be inhibited by Zn^2+^ ions. Showing a highly selective and sensitive turn-on fluorescence in the presence of Zn^2+^, L1 was examined by UV-vis, IR, ^1^H NMR, high resolution electrospray ionization mass spectrometry (HRESIMS), and fluorescence spectroscopy. DFT calculations have been carried out. Apart from the fact that L1 could successfully distinguish Zn^2+^ from Cd^2+^ and Hg^2+^, its potential use in water sample analysis is shown.

## Experimental

### General

All chemicals used were purchased and used as received unless noted otherwise. NMR spectra were taken on a BRUKER Avance FT-NMR AVANCE III (600 MHz). DMSO-d_6_ was used as NMR solvent with chemical shifts (*δ*) being reported in ppm. IR spectra (ATR-IR) were recorded on a BRUKER Alpha T in a range of 400–4000 cm^−1^. Mass spectra were recorded on a BRUKER Impact II mass spectrometer. UV-vis measurements were performed on a JASCO V-550 spectrophotometer. Fluorescence measurements were performed on a JASCO FP-8500 spectrofluorometer using a prismatic cell to avoid inner-field effects. All measurements were conducted at room temperature. The precursor 2-chloro-*N*-(quinolin-8-yl)acetamide was synthesized according to known literature procedures.^26^

### Preparation of 2-((5-methoxy-1*H*-benz[*d*]imidazol-2-yl)thio)-*N*-(quinolin-8-yl)acetamide L1

A sample of 127 mg (0.58 mmol) of 2-chloro-*N*-(quinolin-8-yl)acetamide, 80 mg (0.58 mmol) of potassium carbonate and 104 mg (0.58 mmol) of 5-methoxy-2-mercaptobenzimidazole was dissolved in 5 mL of acetone and refluxed for three hours. Upon completion, monitored by tlc, the reaction mixture was filtered and the solvent was removed *in vacuo* to afford 191 mg of a light brown solid in 91% yield, mp 180 °C. ^1^H NMR (600 MHz, DMSO-d_6_) of a concentrated solution: *δ* = 12.56 (br s, 1 H, –NH), 11.20 (s, 1 H, –NH), 8.80 (dd, *J* = 1.7, 4.2 Hz, 1 H, 2-H), 8.65 (dd, *J* = 1.3, 7.8 Hz, 1 H, 7-H), 8.35 (dd, *J* = 1.7, 8.3 Hz, 1 H, 4-H), 7.64 (dd, *J* = 1.3, 8.3 Hz, 1 H, 5-H), 7.59 (dd, *J* = 4.2, 8.3 Hz, 1 H, 3-H), 7.54–7.57 (m, 1 H, 6-H), 7.40 (d, *J* = 8.7 Hz, 1 H, 7′-H), 7.02 (d, *J* = 2.7 Hz, 1 H, 4′-H), 6.77 (dd, *J* = 2.7, 8.7 Hz, 1 H, 6′-H), 4.33 (s, 2 H, CH_2_), 3.77 (s, 3 H, OCH_3_) ppm. ^13^C NMR (150 MHz, DMSO-d_6_): *δ* = 167.4 (o, C=O), 155.4 (o, 5′-C), 148.8 (+, 2-C), 148.5 (o, 2′-C), 139.8 (o, 3a′-C), 138.1 (o, 8a-C), 136.4 (+, 4-C), 134.8 (o, 7a′-C), 134.5 (o, 8-C), 127.8 (o, 4a-C), 126.9 (+, 6-C), 122.0 (+, 3-C), 122.0 (+, 5-C), 116.3 (+, 7-C), 114.9 (+, 7′-C), 110.5 (+, 6′-C), 97.1 (+, 4′-C), 55.5 (+, OCH_3_), 35.7 (-, CH_2_) ppm. IR (ATR): 3202, 3008, 2947, 2825, 2191, 1660, 1628, 1594, 1522, 1485, 1445, 1426, 1403, 1358, 1340, 1322, 1301, 1264, 1244, 1225, 1202, 1153, 1106, 1087, 1064, 1030, 967, 947, 875, 821, 788, 739, 697, 642, 618, 584, 541, 516, 473, 436 cm^−1^. HR-ESI-MS: calcd. for C_19_H_16_N_4_O_2_S [M + Na]^+^: 387.0892, found 387.0864.

### Fluorescence experiments with various metal ions

9 μL of a 10 mM solution of L1 (0.01 mmol in 1 mL of MeOH) were added to 2.991 mL of MeOH to make a final concentration of 30 μM. Afterwards, 30 μL of a 30 mM MCl_x_-solution (M = K^+^, Na^+^, Ba^2+^, Mg^2+^, Hg^2+^, Cu^2+^, Ca^2+^, Co^2+^, Cd^2+^, Ni^2+^, Al^3+^, Zn^2+^, 0.03 mmol in 1 mL of H_2_O) were titrated to the aforementioned solution of L1. After shaking the sample for a couple of seconds, the fluorescence spectra were measured.

### UV-vis titration experiments

9 μL of a 10 mM solution of L1 (0.01 mmol in 1 mL MeOH) were added to 2.991 mL of MeOH to make a final concentration of 30 μM. Afterwards, 0.50–6.00 μL of a 30 mM Zn-solution (0.03 mmol in 1 mL of H_2_O) were titrated gradually to the aforementioned solution of L1. After shaking the sample for a couple of seconds, the UV-vis spectra were taken.

### Fluorescence titration experiments

9 μL of a 10 mM solution of L1 (0.01 mmol in 1 mL of MeOH) were added to 2.991 mL of MeOH to make a final concentration of 30 μM. Afterwards, 0.25–6.00 μL of a 30 mM Zn solution (0.03 mmol in 1 mL of H_2_O) were titrated gradually to the aforementioned solution of L1. After shaking the sample for a couple of seconds, the fluorescence spectra were measured.

### Competition experiments with various metal ions

9 μL of a 10 mM solution of L1 (0.01 mmol in 1 mL of MeOH) were added to 2.991 mL of MeOH to make a final concentration of 30 μM. Afterwards, 30 μL of a 30 mM MCl_*x*_-solution (M = K^+^, Na^+^, Ba^2+^, Mg^2+^, Hg^2+^, Cu^2+^, Ca^2+^, Co^2+^, Cd^2+^, Ni^2+^, Al^3+^, 0.03 mmol in 1 mL H_2_O) were titrated to the aforementioned solution of L1 followed by the addition of 30 μL of a ZnCl_2_ solution. After shaking the sample for a couple of seconds, the fluorescence spectra were taken.

### Job plot measurement

90 μL of a 10 mM solution of L1 were added to 29.91 mL of MeOH to make a final concentration of 30 μM. This procedure was repeated for ZnCl_2_. Then, 2.7, 2.4, 2.1, 1.8, 1.5, 1.2, 0.9, 0.6, and 0.3 mL of L1 were transferred to individual vials. Afterwards, 0.3, 0.6, 0.9, 1.2, 1.5, 1.8, 2.1, 2.4 and 2.7 mL of the Zn^2+^ solution were added separately to yield a total volume of 3 mL. After shaking the sample for a couple seconds, the fluorescence spectra were taken.

### NMR experiments

Samples of L1 in presence of different equivalents of anhydrous ZnCl_2_ (0.5, 1.0, 2.5 eq.) were dissolved in DMSO-d_6_. Afterwards their ^1^H NMR spectra were measured.

### pH experiments

A series of MeOH : H_2_O (95 : 5, v/v) samples at different pH values were prepared by addition of dilute NaOH or HCl. After the desired pH value was set, 9 μL of L1 were added to 2.991 mL of pH-adjusted MeOH : H_2_O to make a 30 μM concentration. Afterwards, 6.0 μL of a 10 mM ZnCl_2_-solution (0.03 mmol in 1 mL H_2_O) were added to the aforementioned sample. After shaking the sample for a couple of seconds, the fluorescence spectra were taken.

### Theoretical calculations

DFT calculations were performed using ORCA 5 of Neese and co-workers.^27–29^ This DFT package was run on a MS Windows 10 Pro based (Version 21H1) PC system equipped with an AMD Ryzen Threadripper 3970X 32-Core and 128 GB RAM in combination with the appropriate message passing interface MS-MPI 10.0.12498.5. MMFF optimized structures were used as starting geometries for the geometry optimizations with the recently published robust “Swiss army knife” composite method r^2^SCAN-3c of Grimme and co-workers^30^ with D4 dispersion correction and geometrical counter poise correction applying the modified triple-zeta basis set def2-mTZVPP. Subsequent frequency calculation of the final structure evidenced the absence of imaginary frequencies and thus the presence of true minima on the potential energy surface. In case of calculations that include a solvent, the Conductor-like Polarizable Continuum Model (CPCM) implemented in ORCA 5 was applied.

### NMR calculations

Additionally, DFT calculated anisotropic NMR shifts of tautomers L1A and L1B were obtained by means of SPARTAN'20 (www.wavefun.com) with the implemented NMR calculation method based upon the hybrid density functional with dispersion correction ωB97X-D by Chai and Head-Gordon^31^ and the standard basis set 6-31G*. The calculation software was run on the abovementioned MS Windows 10 Pro PC system equipped with the AMD Ryzen Threadripper 3970X 32-core and 128 GB RAM.

### Determination of Zn^2+^ in water samples

An artificially polluted water sample was added to a 30 μM solution of L1 in MeOH, which was prepared as aforementioned. After shaking the sample for a couple of seconds, the fluorescence spectra were taken.

## Results and discussion

**Scheme 1:**
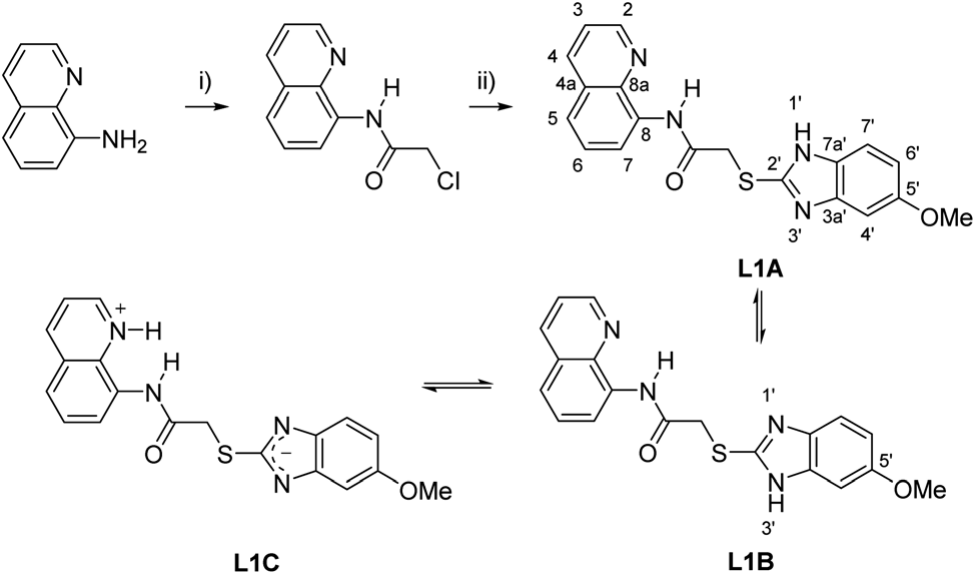
Synthetic route to novel sensor L1 based on 8-aminoquinoline. (i) chloroacetyl chloride, acetone, 0 °C – Rf, 3 h (ii) (a) 5-methoxy-2-mercaptobenzimidazole, potassium carbonate, acetone, Rf, 3 h, 91% yield. Tautomeric structures L1A, L1B and L1C.

The synthesis of the precursor 2-chloro-*N*-(quinolin-8-yl)acetamide and the subsequent reaction with 5-methoxy-2-mercaptobenzimidazole in acetone yielded the desired chemosensor L1 in 91% yield (Scheme 1).

### Tautomerism and hydrogen bonds of L1

**Fig. 1:**
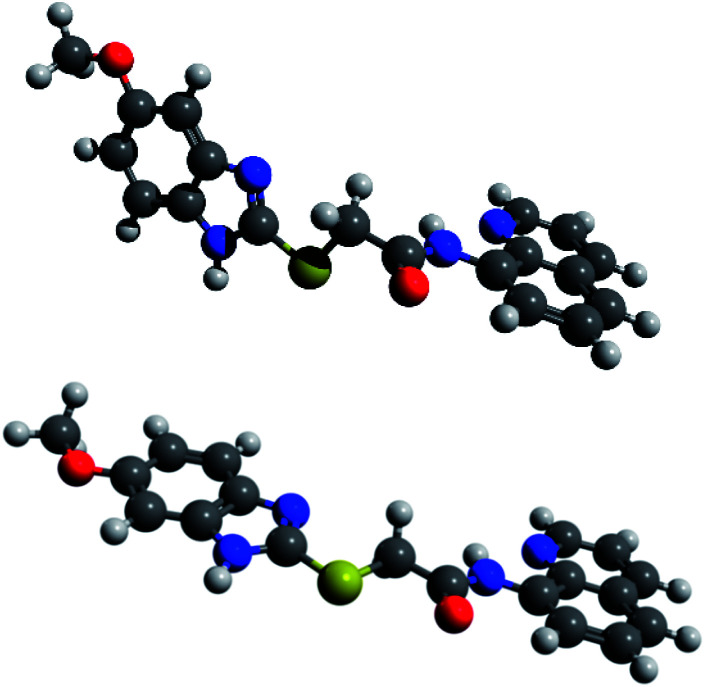
DFT-calculated tautomers L1A (above) and L1B (below) in DMSO.

The structure of L1 enables the formation of tautomers such as L1A–L1C (Scheme 1). In order to elucidate the structure of the predominant tautomers in DMSO, ^1^H NMR and ^13^C NMR studies were performed with concentrated as well as diluted solutions of L1 in DMSO-d_6_. In concentrated solutions, the N–H resonance frequencies of the amide as well as of H–N1′/H–N3′ were observed as extremely broadened signals in the ^1^H NMR spectra. Under these conditions, the chemical shift of 2-H of the quinoline ring of L1 resonates at *δ* = 8.80 ppm in DMSO-d_6_ so that the contribution of the zwitterionic tautomer L1C can be neglected under these conditions. The hydrogen 2-H of quinolinium salts usually resonates at lower fields which was proven by signals of H-2 at *δ* = 8.93 ppm after the addition of gaseous HCl to a solution of L1 in DMSO-d_6_. In accordance with the fact that tautomerization of imidazoles commonly leads to very broad and weak signals in the ^13^C NMR spectra which cannot be detected under standard measurement conditions,^32^ the detection of the ^13^C NMR resonance frequencies of the benzimidazole carbon atoms C-3a′, C-7a', C-7′ and C-4′ required long-term measurements (Fig. S5†). The predominant formation of the two tautomers L1A and L1B was then proven by NMR experiments with diluted solutions in DMSO-d_6_. Under these conditions two distinct sets of benzimidazole protons in addition to the NH resonance frequencies were detectable (Fig. S10–S12†). Full assignment of both tautomers was possible by means of ^1^H, ^13^C-HMBC measurements, especially based on the remarkable carbon shift differences between the adjacent quaternary carbon atoms 3a' and 7a' of the benzimidazole unit. Thus, the signals of tautomer L1A shows a larger shift difference Δ*δ* between its C-3a′ and C-7a′ atoms (Δ*δ* = 14.2 ppm) in comparison to L1B (Δ*δ* = 1.6 ppm). The structure elucidation was strongly supported by DFT NMR shift calculations using the ϖB97X-D functional and the 6-31G* standard basis set within the concurrent Spartan'20 software.^31^ An additional shift prediction^33^ also promoted the structural assignment (Table S2†). Measured shift values, DFT calculations, and classical NMR prediction as a tool in widely used chemistry software (ACD) were in very good agreement. Contrary to these interesting shift differences in the benzimidazole subunit, the corresponding NMR resonances of the quinoline part of the tautomers were virtually isochronous. The calculated structure of L1 in DMSO shows transoid amide bonds with respect to NH̲–C=O̲ which are almost coplanar with the quinoline rings, respectively. The conformer of tautomer L1B is calculated to be 1.1 kJ mol^−1^ more stable than the corresponding tautomer L1A. This small difference is reflected experimentally by an almost equalized tautomer ratio (43% L1A : 57% L1B) in the diluted DMSO-d_6_ solution (Fig. 1).

### Selectivity of L1 for metal ions

**Fig. 2:**
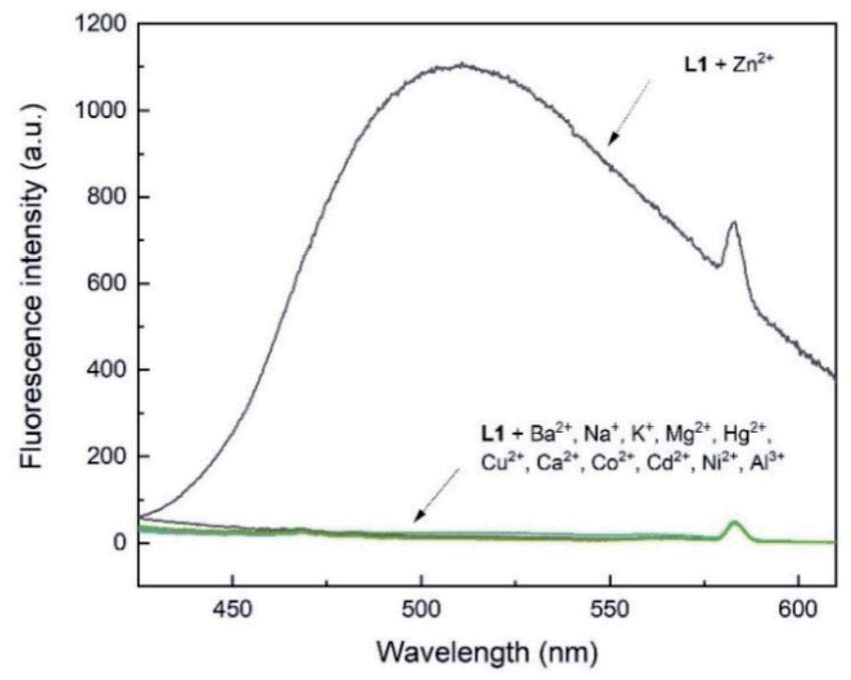
Fluorescence response of L1 (30 μM) in the presence of 10 eq. of various metal ions in MeOH (*λ*_ex_ = 291 nm); metal ions: K^+^, Na^+^, Ba^2+^, Mg^2+^, Hg^2+^, Cu^2+^, Ca^2+^, Co^2+^, Cd^2+^, Ni^2+^, Al^3+^, Zn^2+^. The peak at *λ* = 582 nm results from light reflected of the prismatic cell and equals twice the excitation wavelength.

**Fig. 3:**
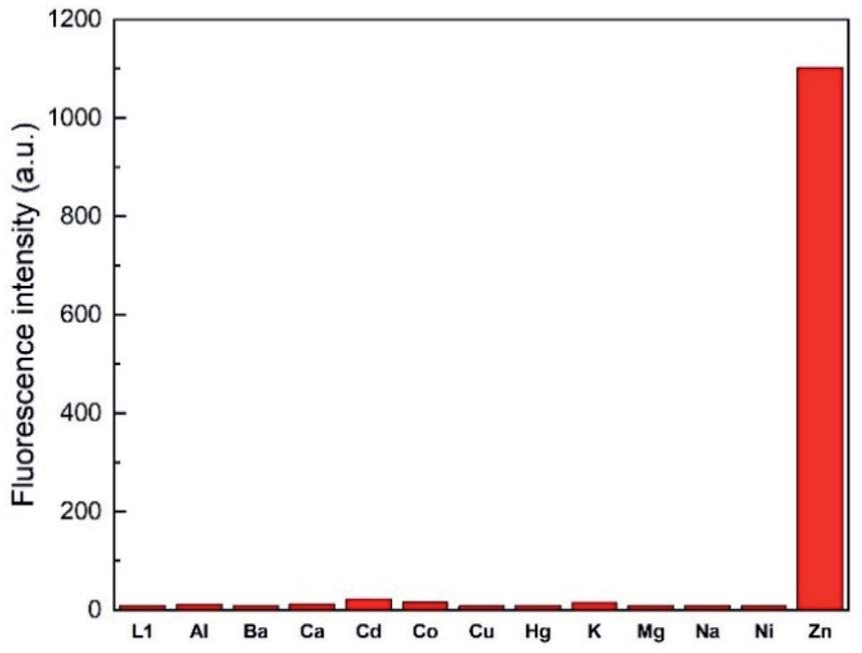
Fluorescence emission intensity of L1 (30 μM) in the presence of 10 eq. of various metal ions in MeOH (*λ*_ex_ = 291 nm).

To assess the selectivity of chemosensor L1, fluorescence spectra in the presence of various metal ions were taken. Herein, K^+^, Na^+^, Ba^2+^, Mg^2+^, Hg^2+^, Cu^2+^, Ca^2+^, Co^2+^, Cd^2+^, Ni^2+^, Al^3+^ and Zn^2+^ were examined (Fig. 2 and 3). The measurements were conducted at an excitation wavelength of *λ*_ex_ = 291 nm. For a better comparability, this average value was determined from isobestic points and absorbance maxima of various structural analogues that we conduct research on. It is evident that chemosensor L1 shows no visible fluorescence in methanol under the measurement conditions. However, upon addition of ten equivalents of Zn^2+^ ions a broad fluorescence band at 510 nm was observed. The narrow peak at 582 nm results from light reflected from the hypotenuse of the prismatic cell with twice the wavelength of the excitation light. In contrast to Zn^2+^ ions, no significant changes in the fluorescence behavior were observed when other metal ions were present. This indicated that L1 is not only suitable as a selective turn-on detector for Zn^2+^ ions, but also suitable for distinguishing Zn^2+^ from metal ions of the same group, *i.e.* Hg^2+^ and Cd^2+^.

### Binding properties of L1

**Fig. 4:**
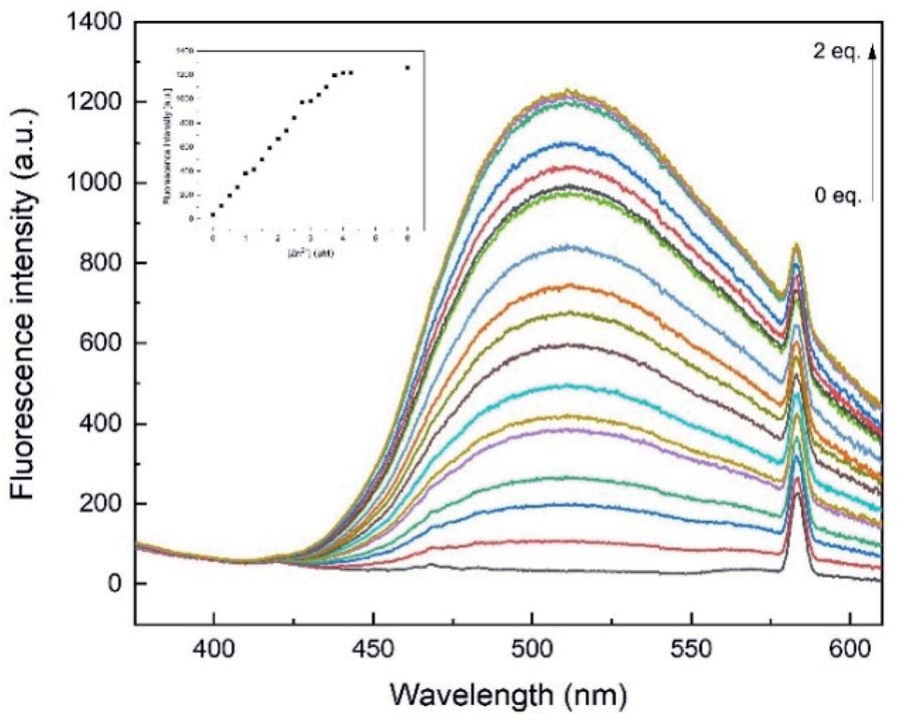
Fluorescence emission spectra of L1 (30 μM) with Zn^2+^ (0–2 eq.) in MeOH (*λ*_ex_ = 291 nm). Inset: plot of the fluorescence intensity at 510 nm as a function of Zn^2+^ concentration. The peak at *λ* = 582 nm results from light reflected of the prismatic cell and equals twice the excitation wavelength.

To examine the binding properties of L1, titration experiments were conducted *via* fluorescence and UV-vis spectroscopy. Fluorescence titration experiments have shown, that upon incremental addition of Zn^2+^, an increasing turn-on fluorescence was observed at 510 nm (*λ*_ex_ = 291 nm, Fig. 4). Chemosensor L1 showed no fluorescence at 510 nm, which might be due to a photo-induced electron transfer (PET) to the quinoline moiety induced by benzimidazole nitrogen atoms.^34^ As shown in Fig. 2, a strong fluorescence enhancement was observed when Zn^2+^ was added which can be attributed to a chelation-induced enhanced fluorescence (CHEF).^35^ Further addition of Zn^2+^ past 1 eq. did not cause significant changes regarding the fluorescence intensity.

**Fig. 5:**
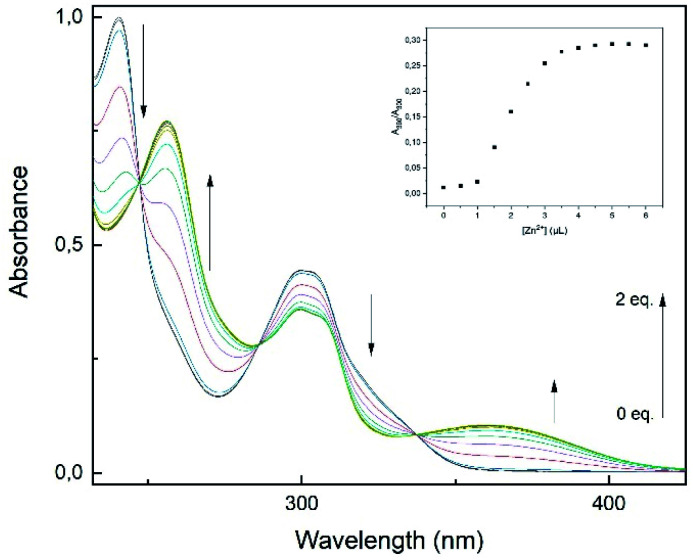
UV-vis titration spectra of L1 (30 μM) with Zn^2+^ (0–2 eq.) in MeOH. Inset: plot of absorption ratio *A*_360_/*A*_300_ as a function of Zn^2+^ concentration.

Additionally, UV-vis experiments have been conducted to further examine the binding properties. The UV-vis absorbance spectra of L1 (30 μM) in methanol display two distinct absorption bands at 241 and 300 nm, respectively (Fig. 5). These bands have been assumed to be due to π–π* and n–π* transitions of aminoquinolines.^35^

Additionally, these absorption bands redshifted to 256 and 360 nm accompanied by three isobestic points at 247, 286 and 337 nm. The spectral response suggests the formation of only one L1–Zn-complex.^12,21,35^ Furthermore, the incremental addition of Zn^2+^ (0–2 eq.) showed saturation at a L1–Zn-ratio of 1 : 1, as the absorption ratio *A*_360_/*A*_300_ did not change significantly after 1 eq. (Fig. 5, inset). The results derived from the titration experiments indicated that formation of a 1 : 1 complexation must be the case.

### Job plot and Benesi–Hildebrand analysis

**Fig. 6:**
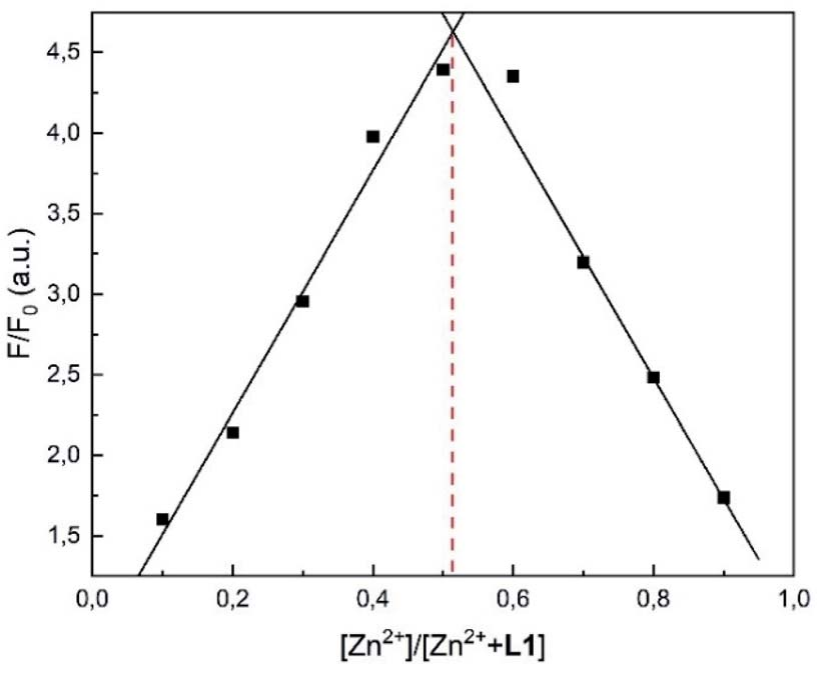
Job plot analysis using the fluorescence intensity at 510 nm of L1 and Zn^2+^ in MeOH.

**Fig. 7:**
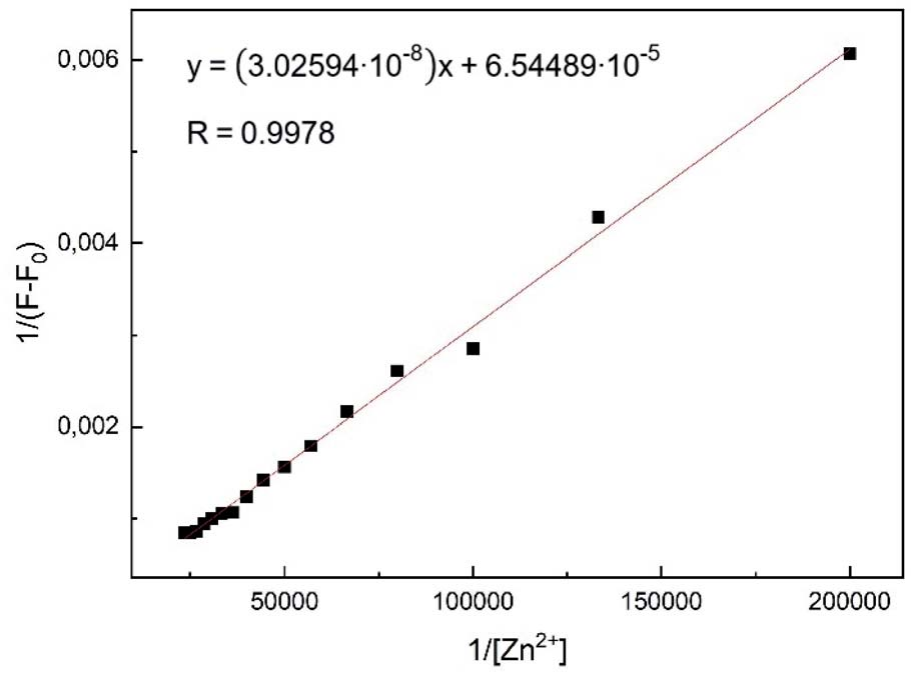
Benesi–Hildebrand plot of L1 in MeOH using eqn (1). Assumed complexation stoichiometry 1 : 1 of L1–Zn^2+^.

In order to verify the stoichiometry, a Job plot analysis was performed.^36^ As seen in Fig. 6, the emission maximum was observed at a molar fraction of 0.5.^8,34^ This indicated that a 1 : 1 complex was formed, which is also visible in the HR-ESI mass spectra (Fig. S1†). The peaks at (*m*/*z*) 427.0199 and 462.9965 were attributed to [L1 + Zn^2+^-1] (calc. 427.0207) and [L1 + Zn^2+^ + Cl^−^] (calc. 462.9973), respectively. The 1 : 1 complexation was further confirmed by the Benesi–Hildebrand method (Fig. 7).^37^

Plotting 1/Δ*F* against 1/[Zn^2+^] yielded a linear regression. Using titration data, the Benesi–Hildebrand equation for 1 : 1 complexes is defined as follows:^38^1
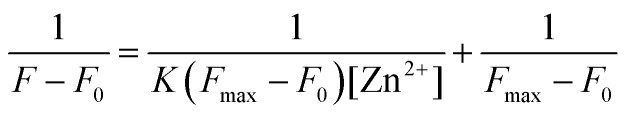


The binding constant was calculated to be *K*_b_ = 2.16 × 10^3^ M^−1^ for the L1–Zn-complex and is in accordance to expected values, according to literature (1–10^12^).^8,39^

### Detection limit

The limit of detection (LOD) was calculated by the equation 3*σ*/*s*.^40^ Herein, *σ* represents the standard deviation of blank measurements and *s* is the slope between the fluorescence intensity and Zn^2+^ concentration (Fig. S3†). The standard deviation *σ* over six blank measurements was calculated to be 1.8678.

According to the equation, the detection limit of L1 was found to be 1.76 × 10^−7^ M, which proved to be much lower than the WHO guideline (76 μM) for Zn^2+^ ions in drinking water.^41^ In comparison to other studies, our determined LOD appeared to be lower than reported Zn^2+^ chemosensors (Table S1†). Additionally, the reversibility of the L1–Zn complex was examined. Upon addition of excess EDTA, the fluorescence emission of the L1–Zn complex was successfully reverted. This proved the reversible use of the synthesized chemosensor L1 (Fig. S2†).

### Competition experiments

**Fig. 8:**
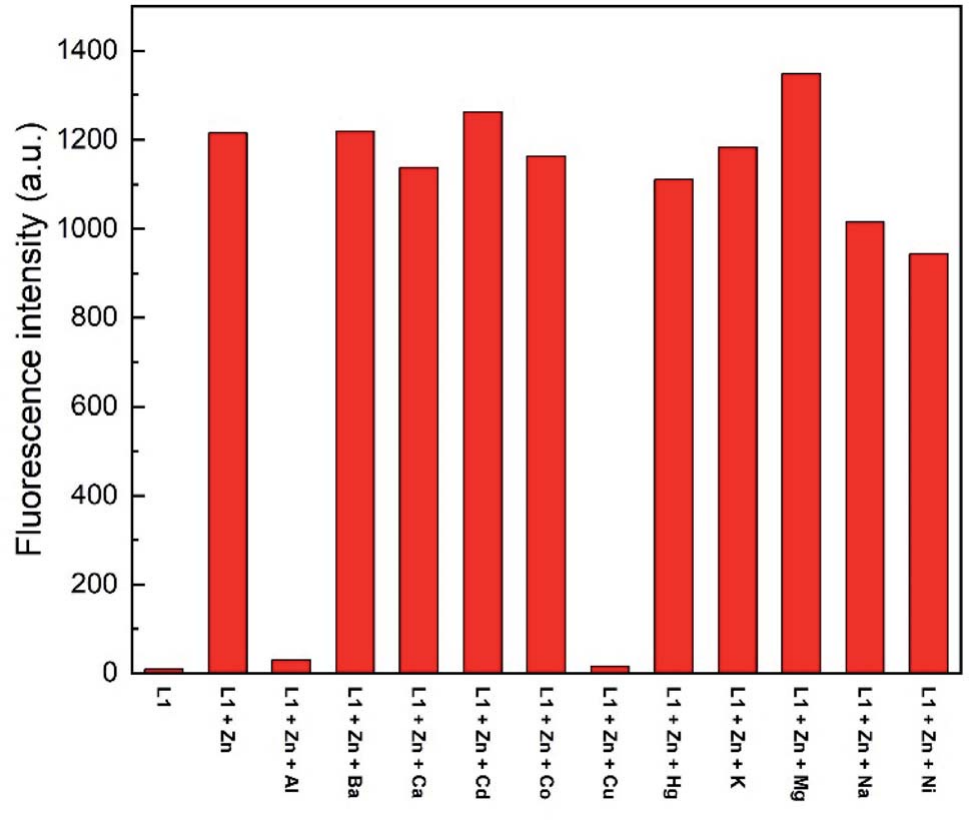
Competition studies of L1 (30 μM) toward Zn^2+^ (10 eq.) in the presence of various metal ions (10 eq.) in MeOH (*λ*_ex_ = 291 nm).

In order to examine the effect of other cations on the fluorescence emission of the L1–Zn complex, competition experiments were conducted (Fig. 8). In presence of 10 equivalents of Zn^2+^ cations various metal ions have been added to the L1 sample. Ba^2+^, Ca^2+^, Co^2+^ and K^+^ ions have proven to show no effect, whereas Na^+^ and Ni^2+^ caused negligible fluorescence quenching to the L1–Zn complex. The presence of same group metal ions, Cd^2+^ and Hg^2+^, caused no interference to the fluorescence emission induced by Zn^2+^. This additionally proved that chemosensor L1 can easily distinguish Zn^2+^ from Cd^2+^ and Hg^2+^. However, a strong quenching phenomenon was observed in the presence of both Al^3+^ and Cu^2+^ ions. It is known that Zn^2+^ detection can be quenched in the presence of Cu^2+^ and that a cation–exchange reaction between zinc and metal ions such as Al^3+^ can take place.^19,42^ The Zn-selective behaviour can be explained through Pearson's HSAB model.^43^ Due to the harder nature of the incorporated oxygen and nitrogen atoms, it is evident that Zn^2+^, Cu^2+^ and Al^3+^, as harder metal centres, preferably interact with these receptor sites. Furthermore, it has been reported that the incorporation of nitrogen and oxygen atoms into ligand systems has proven to favour the complexation of Zn^2+^ ions in contrast to other metal ions.^44^

### pH experiments

**Fig. 9:**
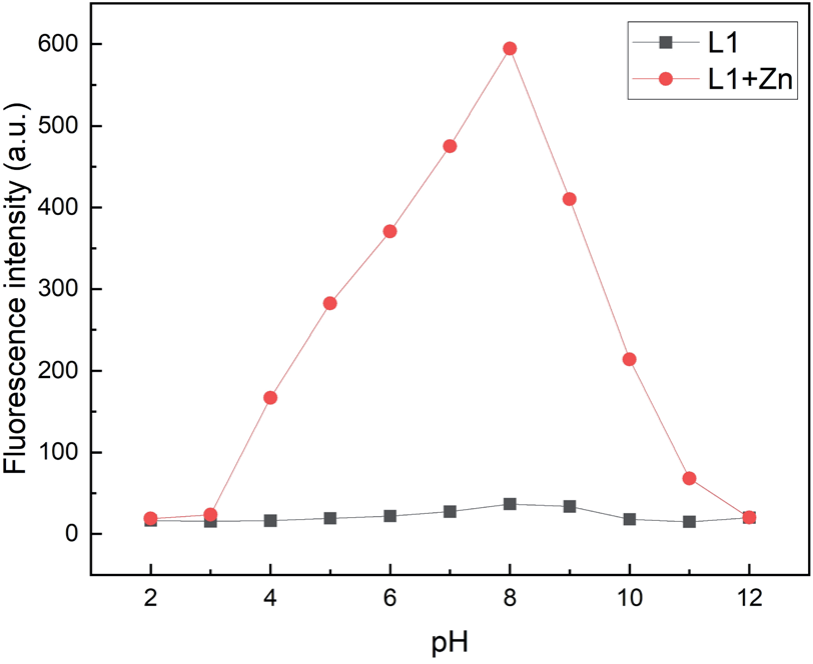
Fluorescence intensities (*λ*_em_ = 510 nm) of L1 (30 μM) in the absence and presence of Zn^2+^ at various pH values in MeOH/H_2_O (95 : 5, v/v) (*λ*_ex_ = 291 nm).

The pH-dependence of various quinoline chemosensors has been reported.^12,45^ To assess the photophysical properties, the fluorescence emission was examined at different pH values in MeOH : H_2_O (95 : 5, v/v). As seen in Fig. 9, L1, in presence of Zn^2+^, exhibits the strongest fluorescence emission at a pH value of 8. In contrast, under strongly acidic or basic conditions a considerable fluorescence quenching is observed. At low pH values, this might be attributed to possible protonation of nitrogen sites in quinoline or benzimidazole moieties.^46^ Fluorescence quenching at higher pH values might be due to the deprotonation of NH fragments resulting in a stronger PET towards the fluorophore. Nevertheless, in the pH range from 4 to 10 L1 exhibits a satisfactory fluorescence response with a peak at a pH value of 8, thus demonstrating that the detection of Zn is possible under physiological pH conditions.

### Complexation studies

**Fig. 10:**
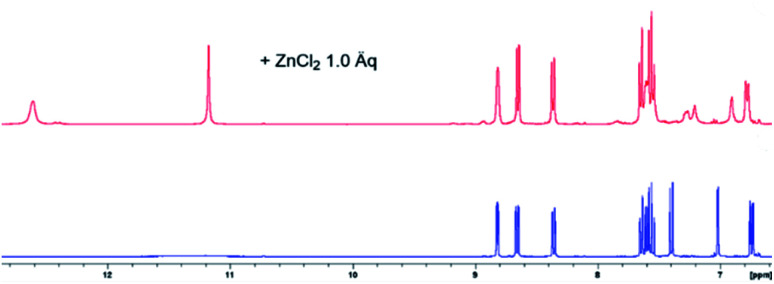
Aromatic proton shift of L1 (blue, concentrated solution in DMSO-d_6_), upon addition of 1 eq. ZnCl_2_ (red). The addition past 1 eq. of Zn^2+^ yielded no further changes (Fig. S7†).

NMR spectroscopic analyses were finally conducted to investigate the binding behaviour of L1 in presence of Zn^2+^ ions (Fig. 10). Upon addition of Zn^2+^ to the concentrated solution of L1 in DMSO-d_6_, two distinct sharp –NH signals were observed at 12.61 and 11.17 ppm, respectively, indicative of an inhibited tautomerism. Whereas the signals of H-4, H-5, H-6 and H-7 of L1 shifted only slightly on addition of Zn^2+^, the signals of H-2 and H-3 were considerably broadened, hinting at a complexation through the quinoline N-atom. As the benzimidazole protons H-4′ and H-7′ showed significant upfield shifts on complexation with Zn^2+^, one of its N-atoms obviously is involved in complexation [*e.g*. Δ*δ*(H-4′) = 0.11 ppm; *e.g*. Δ*δ*(H-7′) = 0.16 ppm]. The third complexation site can be identified by ^13^C NMR spectroscopy. Thus, the addition of Zn^2+^ ions induced a significant shift of the ^13^C NMR resonance frequencies of the carbonyl carbon atom to higher fields [Δ*δ*(C=O) = 0.159 ppm, Fig. S8†], accompanied by a considerably enlarged peak width at half-height. Significant changes were also observed in case of the signals of the benzimidazole carbon atoms C-5′ and C-6′ (Fig. S9†). IR-spectroscopic investigations unambiguously support the participation of the carbonyl oxygen upon zinc complexation in the solid state. The carbonyl stretching vibration of the free ligand L1 appears at 1661 wavenumbers (ATR IR), whereas this band is shifted to 1595 cm^−1^ in the zinc complex (Fig. S4b†). Actually, in the DFT calculated IR of this complex (functional r^2^SCAN-3c; def2-mTZVPP basis set) this crucial band is found with excellent congruence at 1598 cm^−1^ (Fig S4c†).

We performed comparative DFT calculations of the Zn complex with the recently published r^2^SCAN-3c method of Grimme and co-workers^30^ recently implemented in ORCA 5^27–29^ and additional consideration of DMSO by the CPCM solvent model supported by ORCA.

**Fig. 11:**
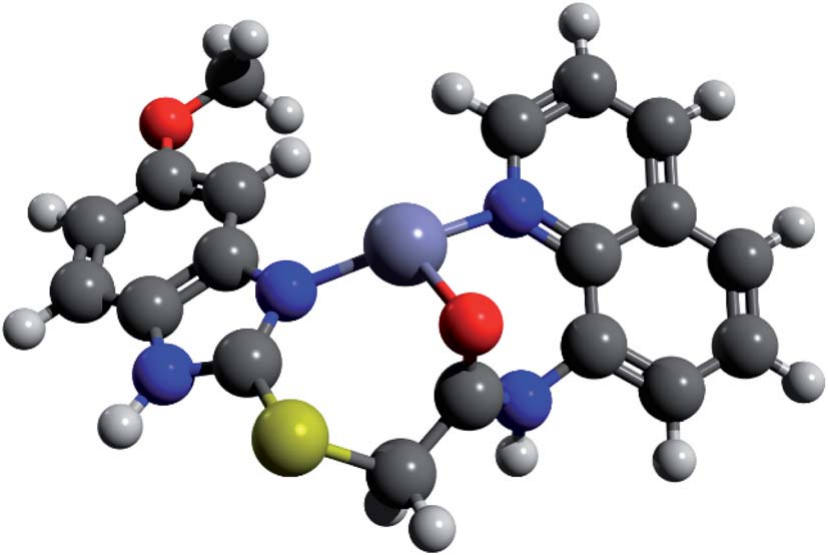
DFT-calculated L1A–Zn complex in DMSO (r^2^SCAN-3c/def2-mTZVPP).

The calculations resulted in a structure of the complex which is in total accordance with the experimental data and which is shown in Fig. 11. Utilizing Pearson's HSAB model,^43^ the sulfur atom was readily ruled out as a potential complexation site. As a borderline metal ion, Zn^2+^ shows a greater affinity towards harder oxygen and nitrogen atoms as opposed to the softer sulphur centre which is in accordance with previous studies.^47^ The zinc complex based on ligand tautomer L1A is energetically favoured with a difference of 3.7 kJ mol^−1^ in comparison to L1B. This is probably caused by the positive mesomeric effect of the methoxy group in 5-position of the benzimidazole moiety that supports the nitrogen donor centre of the benzimidazole moiety. DFT calculations predict that the tautomer L1A is fixed upon addition of Zn^2+^ and the PET to the fluorophore deriving from the nitrogen atom of the benzimidazole moiety is inhibited, resulting in a chelation-induced enhanced fluorescence (CHEF) of L1A–Zn (Fig. S17†).

### Determination of Zn^2+^ in water samples

Finally, the applicability of L1 was tested with water samples. We created artificially polluted water samples by the addition of Ca^2+^, Na^+^, K^+^, Mg^2+^ aside metal ions of the same group, Cd^2+^ and Hg^2+^, to water. Plotting the fluorescence intensity against the Zn^2+^ concentration yielded a linear calibration plot (Fig. S3†) which was used to determine the Zn^2+^ content in given water samples. Table 1 shows that L1 was successfully able to recover the given Zn^2+^ concentrations even in the presence of various metal ions. Therefore, it is safe to assume that L1 could potentially be used for Zn^2+^ detection in real water samples.

**Table 1:** Determination of Zn^2+^ in artificially polluted water samples

Sample	Zn(ii) present (μM L^−1^)	Zn(ii) found (μM L^−1^)	Recovery (%)	R.S.D (*n* = 3)
Sample 1^a^	5.00	5.50	110.0	6.5
Sample 2^a^	10.00	10.48	104.8	2.1

a Aqueous solution. 1/2: 5/10 μmol L^−1^ Zn(ii), 8.5/17 μmol L^−1^ Cd^2+^, Ca^2+^, Hg^2+^, Na^+^, K^+^, Mg^2+^. Conditions for L1 = 30 μM solution in MeOH.

## Conclusions

To summarize, we have designed and synthesized a new chemosensor L1 based on 8-aminoquinoline bearing a benzimidazole moiety. L1 showed a high selectivity and sensitivity towards Zn^2+^ in methanol, which was accompanied by a distinct green fluorescence emission. Moreover, L1 was capable of distinguishing Zn^2+^ from same group metal ions Cd^2+^ and Hg^2+^. The LOD was determined to be 0.176 μM, which proved to be lower than the WHO standard (76 μM). Spectroscopic studies have shown that a 1 : 1 complexation takes place, which upon addition of EDTA showed the possible reversibility of the L1–Zn complex. The prototropic tautomerism exhibited by the benzimidazole moiety was used as proof to successfully identify the binding sites. Furthermore, the capability of L1 to quantify Zn^2+^ in water samples was shown. Hence, we believe that L1 shows a great potential for use in both biological and environmental applications.

## Conflicts of interest

There are no conflicts to declare.

## Supplementary Material

RA-011-D1RA05591G-s001
