# Supplementary material for: Zn2+ detection of a benzimidazole 8-aminoquinoline fluorescent sensor by inhibited tautomerization
Source: RSC Adv. 2021 Nov 11;11(58):36450–8. doi: 10.1039/d1ra05591g (PMC9043439; doi:10.1039/d1ra05591g)
Supplement: RA-011-D1RA05591G-s001 [file RA-011-D1RA05591G-s001.pdf]

Electronic Supplementary Material (ESI) for RSC Advances.
This journal is © The Royal Society of Chemistry 2021

Supporting Information

Zn^{2+} detection of a benzimidazole 8-aminoquinoline fluorescent sensor by inhibited tautomerization

Harun Taş, Jörg Adams, Jan C. Namyslo, Andreas Schmidt*

Contents

1. NMR spectra of L1 2

2. HR-ESI-MS of L1/L1-Zn 4

3. Reversibility of L1-Zn with EDTA 5

4. Plot of Fluorescence intensity vs. Zn^{2+} concentration 6

5. FT-IR spectra of L1 and L1-Zn complex 7

6. ^{13}C-NMR long term measurement 9

7. Benesi-Hildebrand analysis 10

8. ^{1}H-NMR experiments 11

9. ^{13}C-NMR experiments of L1-Zn 12

10. Diluted NMR-sample of L1-tautomers 14

11. DFT Calculations 17

12. LOD-comparison of L1 to other Zn chemosensors (Table S1) 24

13. NMR-calculations of quaternary C atoms/ $\Delta\delta$ determination (Table S2) 25

14. Sources/References 26

1. NMR spectra of L1

^{1}H-NMR of L1

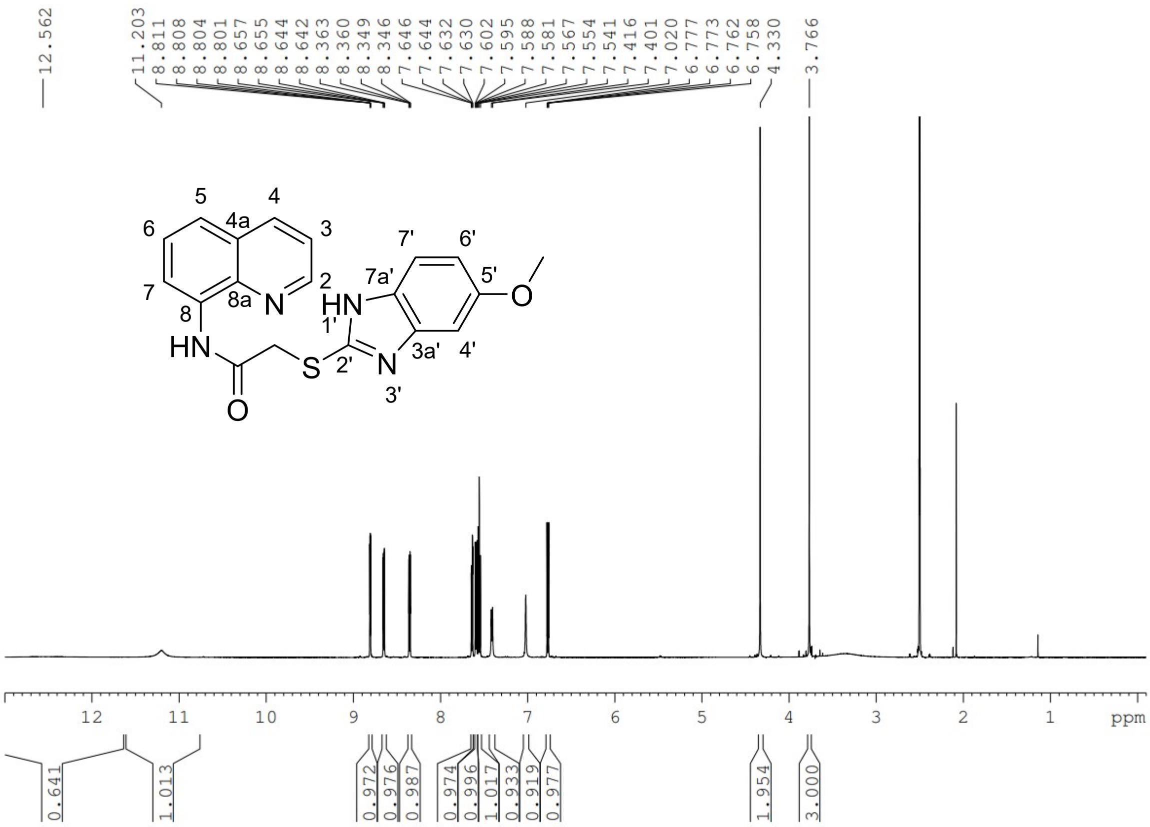

^{13}C-NMR of L1

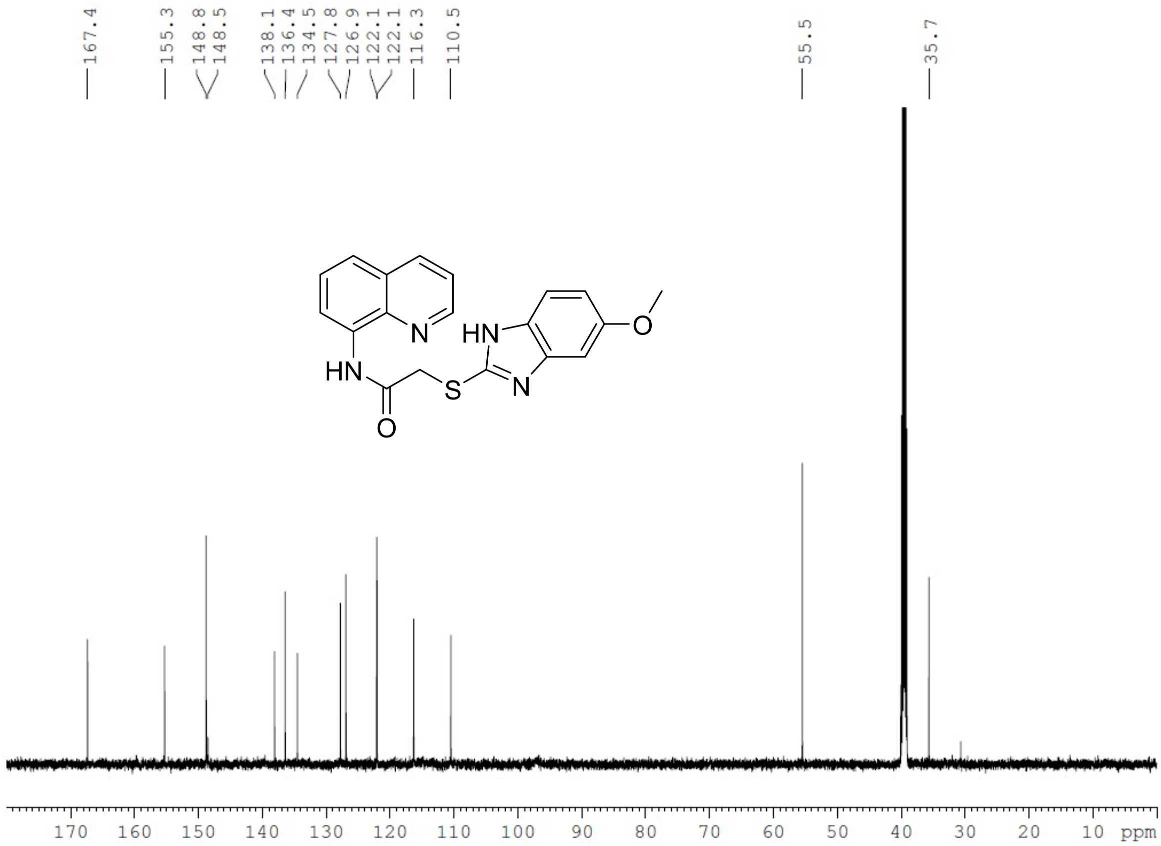
167.4
155.3
148.8
148.5
138.1
136.4
134.5
127.8
126.9
122.1
122.1
116.3
110.5
55.5
35.7
HN
N
S
N
HN
O
O
170 160 150 140 130 120 110 100 90 80 70 60 50 40 30 20 10 ppm

2. HR-ESI-MS of L1/L1-Zn

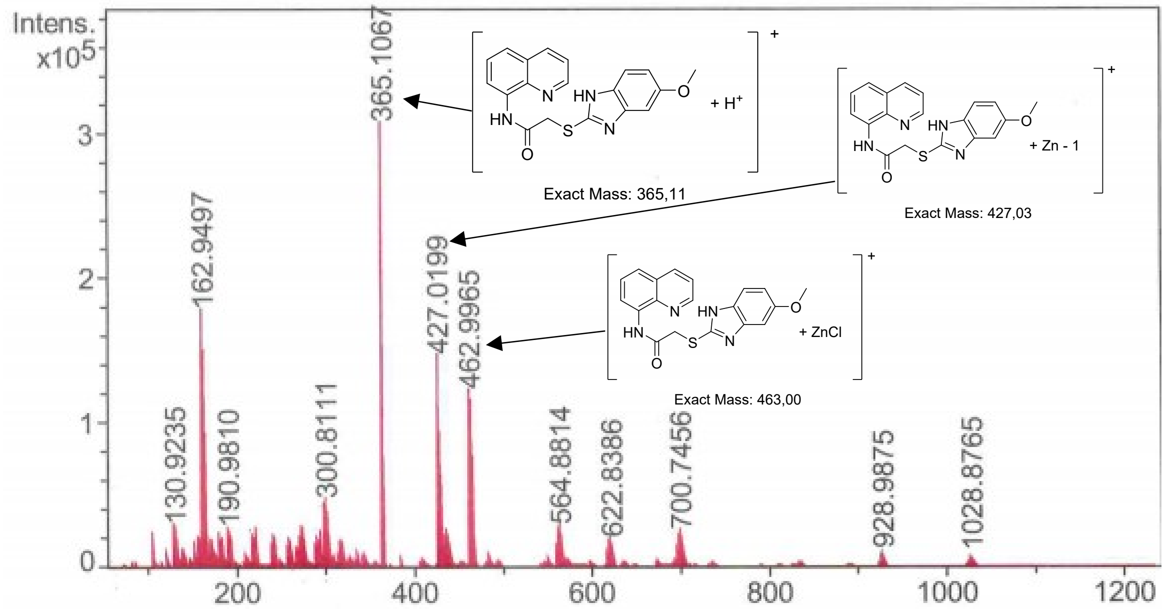

Figure S1: HR-ESI mass spectrum of **L1** upon addition of Zn^{2+}.

3. Reversibility of L1-Zn with EDTA

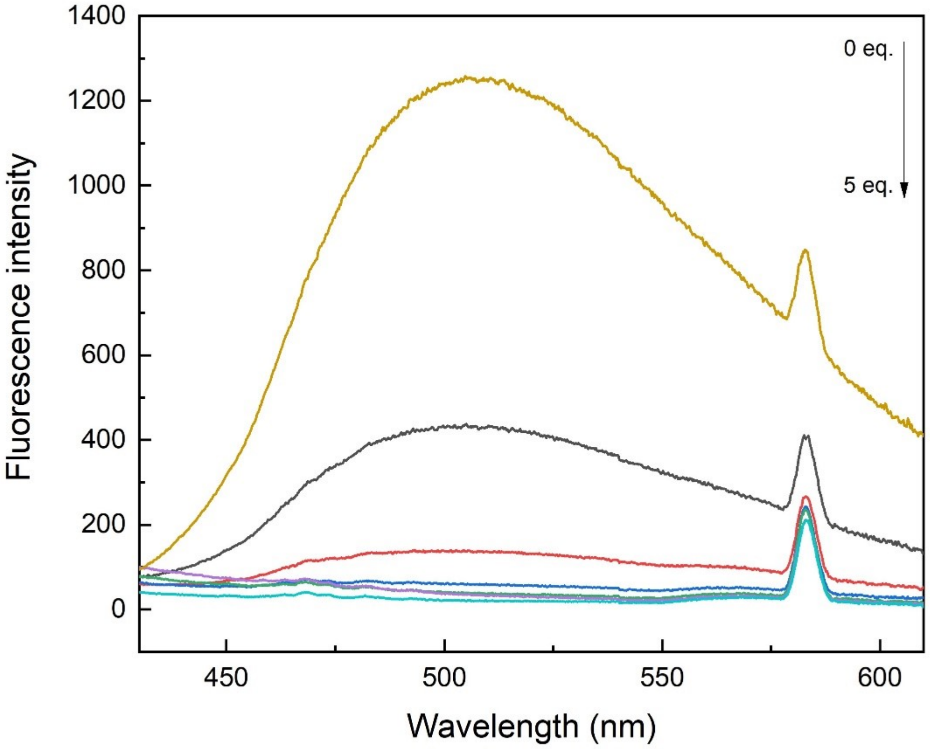

Figure S2: Fluorescence emission spectra of **L1**+Zn^{2+} upon incremental addition of EDTA.

4. Plot of Fluorescence intensity vs. Zn^{2+} concentration

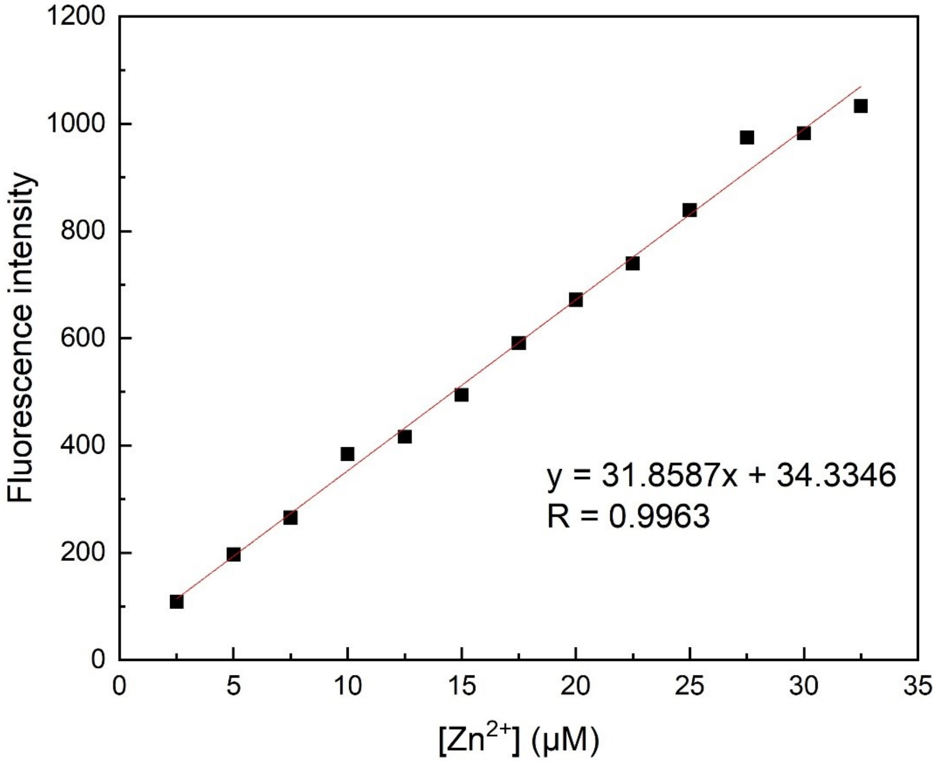

Figure S3: Fluorescence intensity as a function of [Zn^{2+}] concentration. Concentration of **L1**: 30 μM.

5. FT-IR spectra of L1 and L1-Zn complex

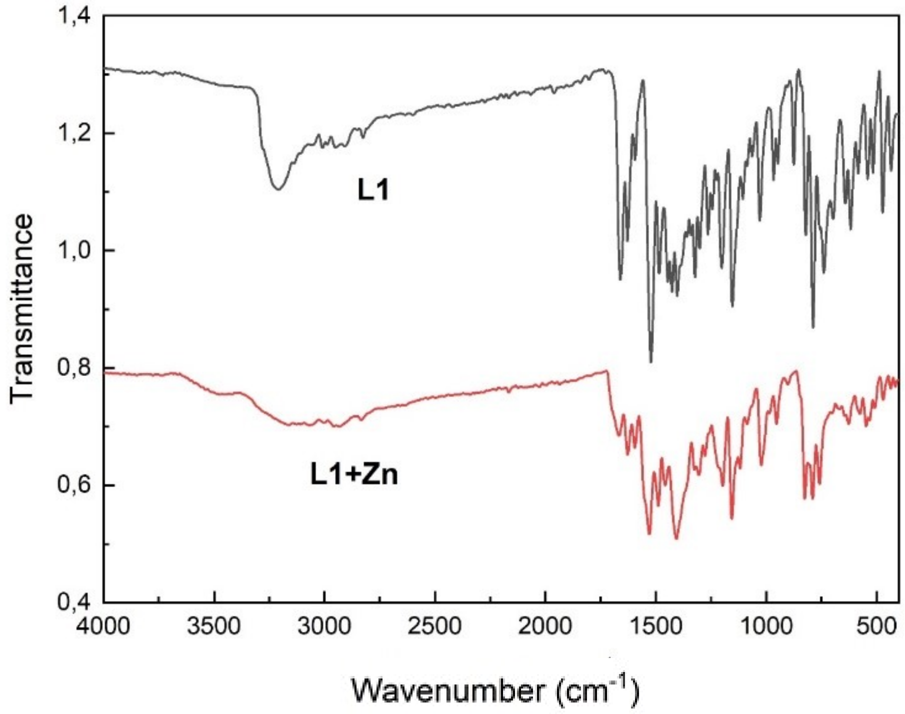

Figure S4a: FT-IR spectrum of **L1** (black) and the **L1**-Zn-complex (red).

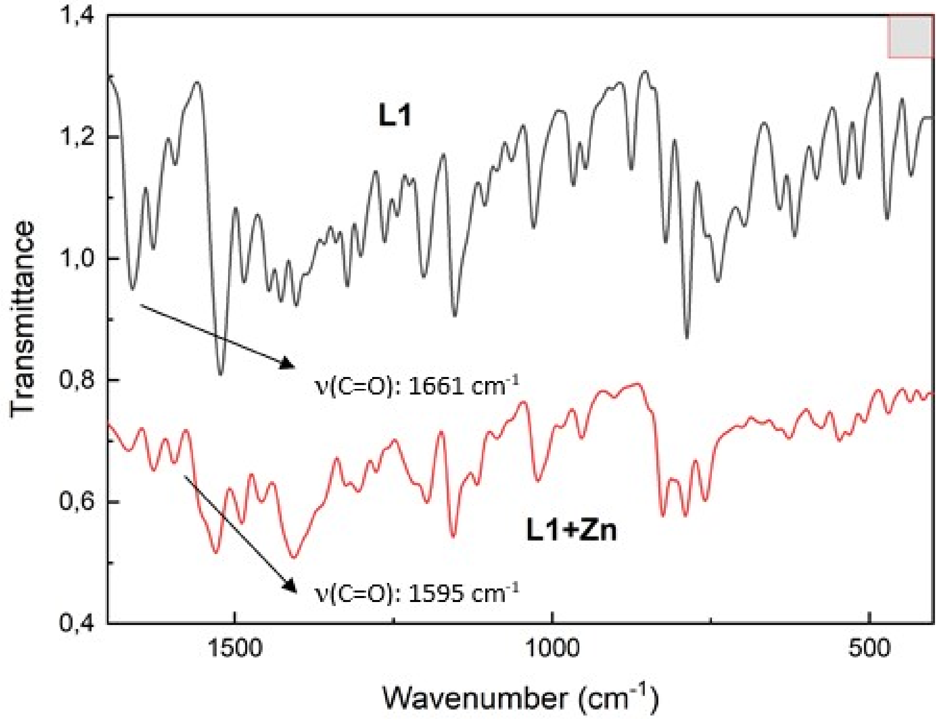

Figure S4b: FT-IR spectrum: 1700 – 400 cm^{-1} area of **L1** (black) and the **L1**-Zn-complex (red).

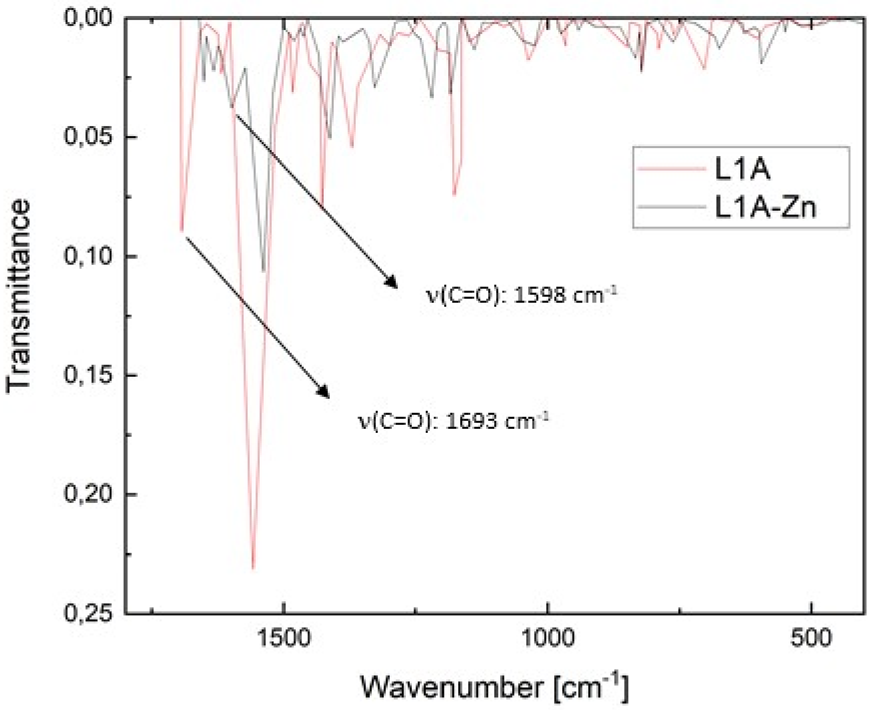

Figure S4c: Calculated FT-IR spectrum: 1700 – 400 cm^{-1} area of **L1** (black) and the **L1**-Zn-complex (red).

6. ^{13}C-NMR long term measurement

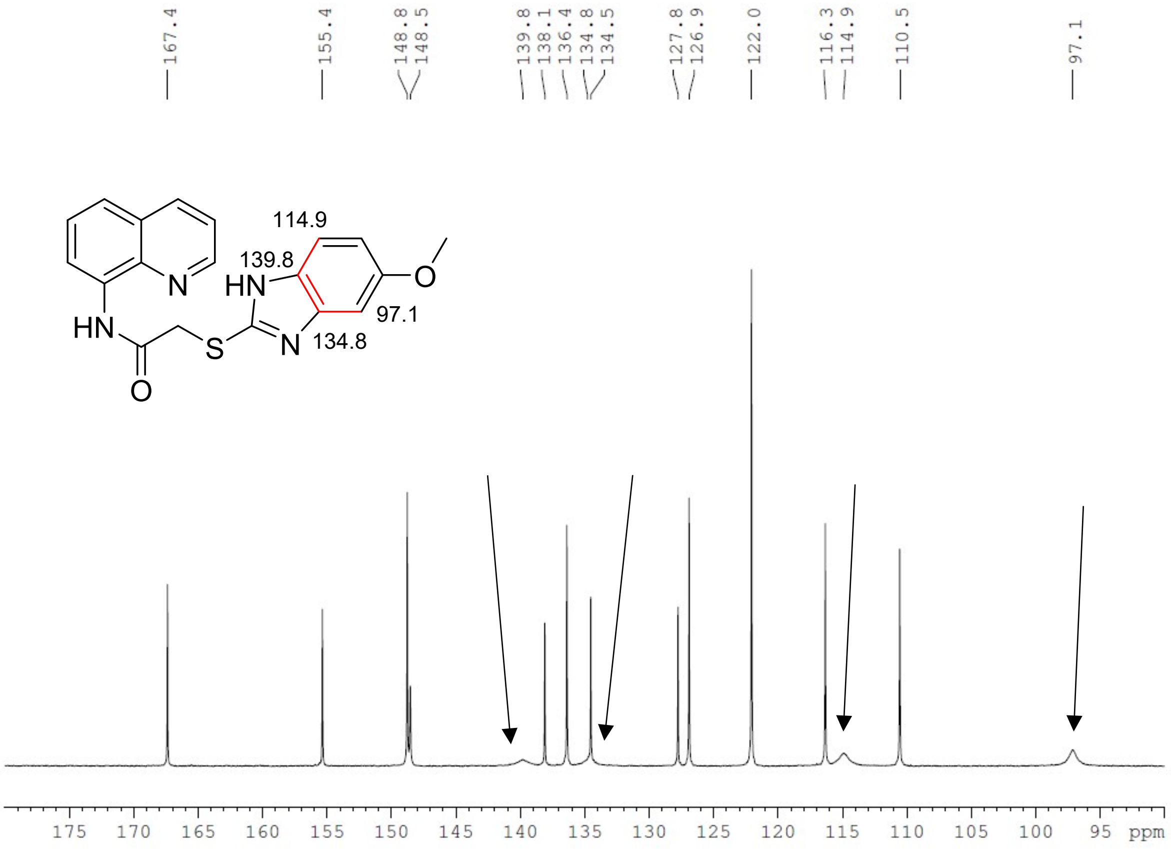

Figure S5: ^{13}C-NMR experiment of **L1** for making C-7a', C-3a', C-7' and C-4' atoms visible.

7. Benesi-Hildebrand analysis

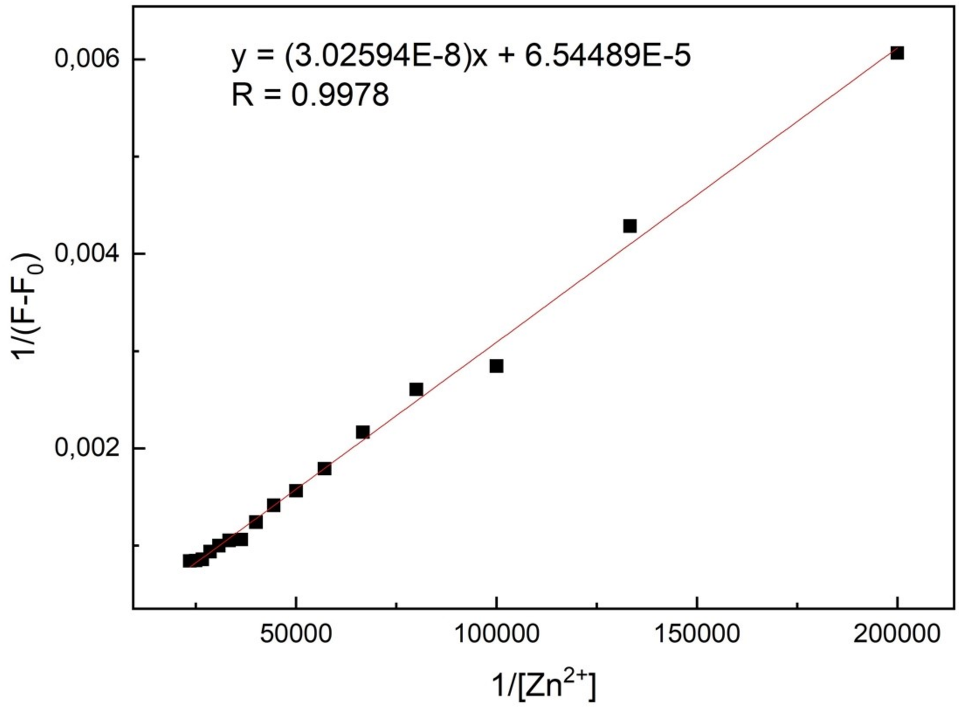

Figure S6: Benesi-Hildebrand plot of **L1** in MeOH using equation (1). Assumed complexation stoichiometry 1:1 of **L1**-Zn^{2+}.

The binding constant for 1:1 complexes was calculated according to (1):[1]

$$\frac{1}{F - F_0} = \frac{1}{K(F_{max} - F_0)[Zn^{2+}]} + \frac{1}{F_{max} - F_0} \tag{1}$$

F resembles fluorescence intensities of **L1** (λ_{max} = 510 nm) in the absence (F_0), in the presence (F) and at a concentration of complete interaction of **L1** with Zn^{2+} (F_{max}). K is the binding constant and [Zn^{2+}] the concentration of added Zn^{2+} ions, respectively.

Plotting 1/(F-F_0) against 1/[Zn^{2+}] yields a linear plot with an equation of y = *b* x + *a*. Therefore, the following can be assumed for the intercept *a*, the slope *b* and the binding constant *K*:

$$a = \frac{1}{F_{max} - F_0}, \quad b = \frac{1}{K(F_{max} - F_0)}, \quad K = \frac{a}{b} = \frac{6.54489 \cdot 10^{-5}}{3.02594 \cdot 10^{-8}} = 2.16 \cdot 10^{3}\ (mol^{-1} \cdot L)$$

8. ¹H-NMR experiments

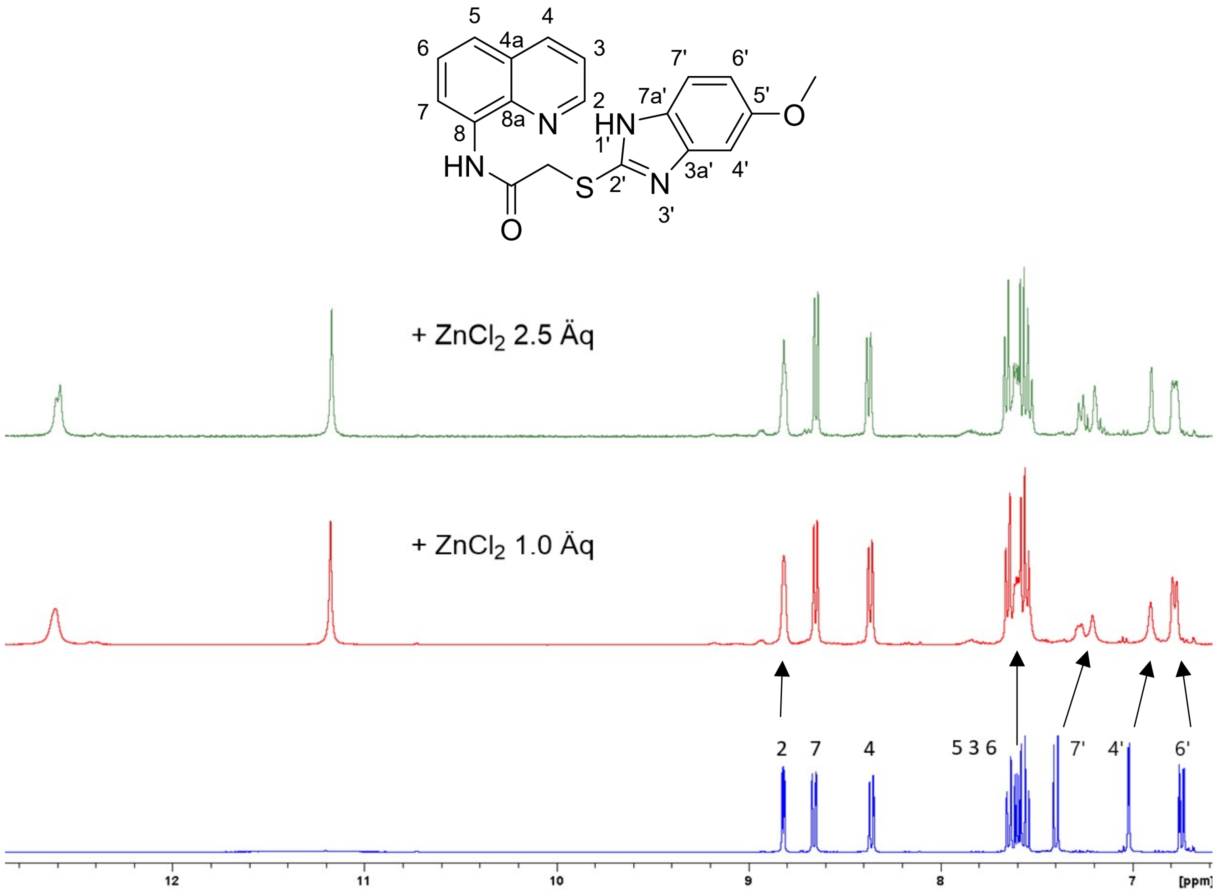

Figure S7: ¹H-NMR titration experiments of **L1** with Zn^{2+}.

9. ^{13}C-NMR experiments of L1-Zn

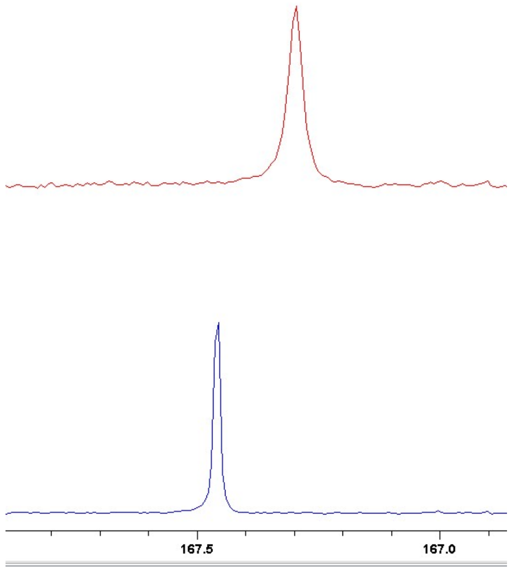

Figure S8: Carbonyl fragment of **L1** (blue) and of **L1-Zn** complex (red).

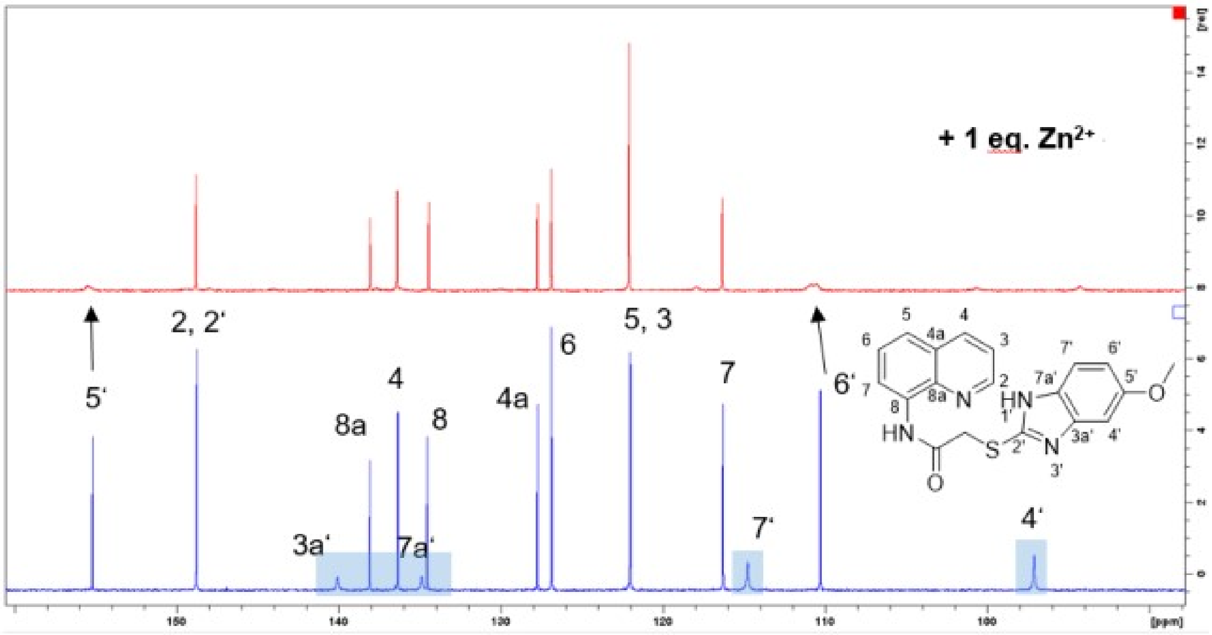

Figure S9: Comparison of ^{13}C NMR spectra of **L1** and **L1**-Zn, shown: aromatic carbon atoms. Adjacent carbon atoms to the tautomer centre marked in blue.

10. Diluted NMR-sample of L1-tautomers

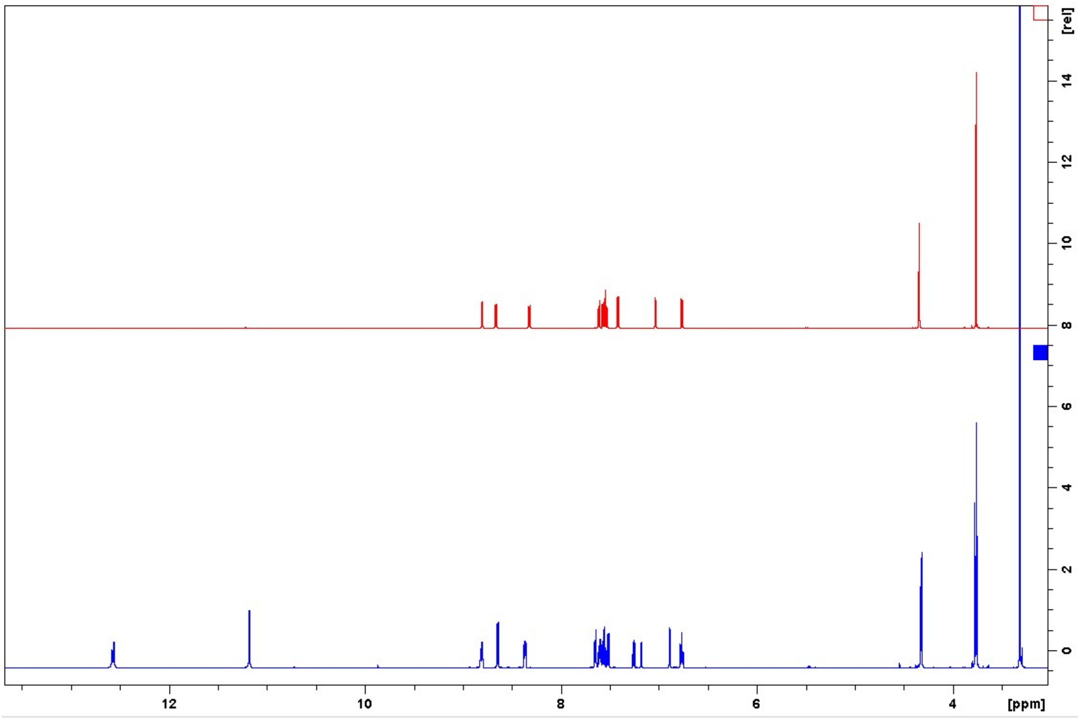

Figure S10: Comparison of ^{1}H NMR spectra of a diluted (blue) and a regular (red) **L1** sample in DMSO-d_6.

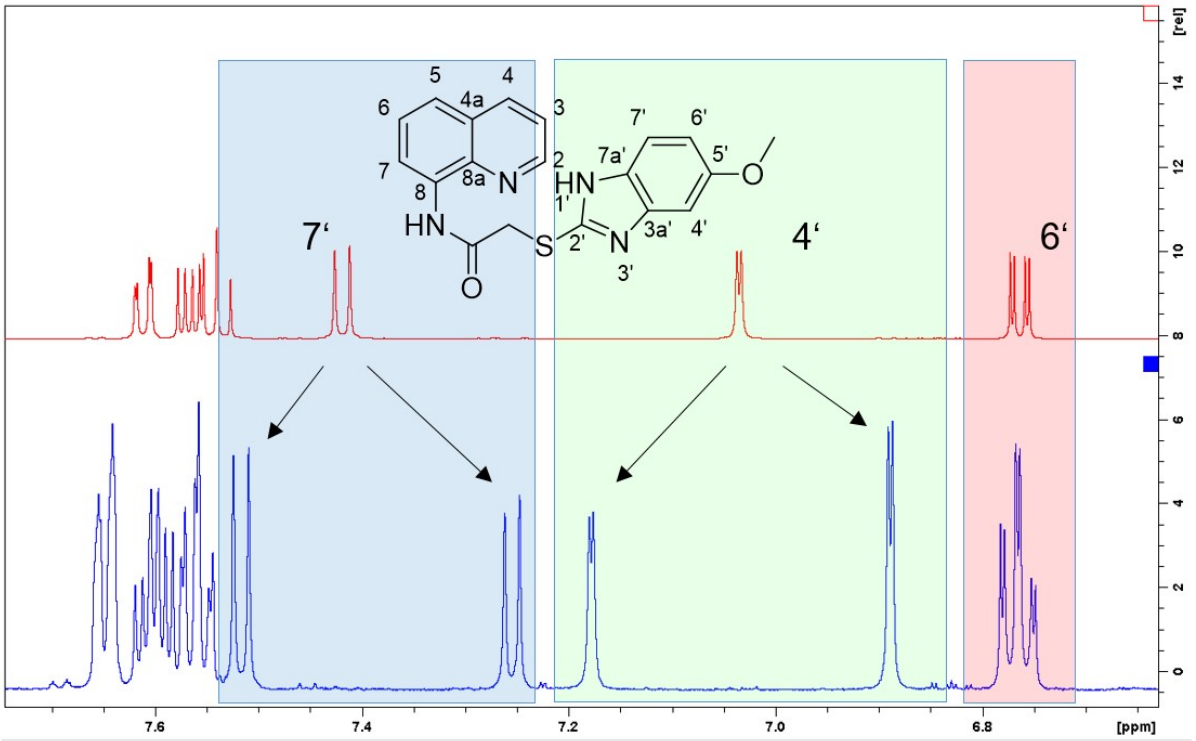

Figure S11: ^{1}H NMR spectroscopic comparison of the benzimidazole proton shifts of a diluted (blue) and a regular (red) **L1** sample in DMSO-d_6.

Carbon atom shifts were calculated using the ACD/C+H NMR Predictors and DB 2018.2.5 software (shown in red).

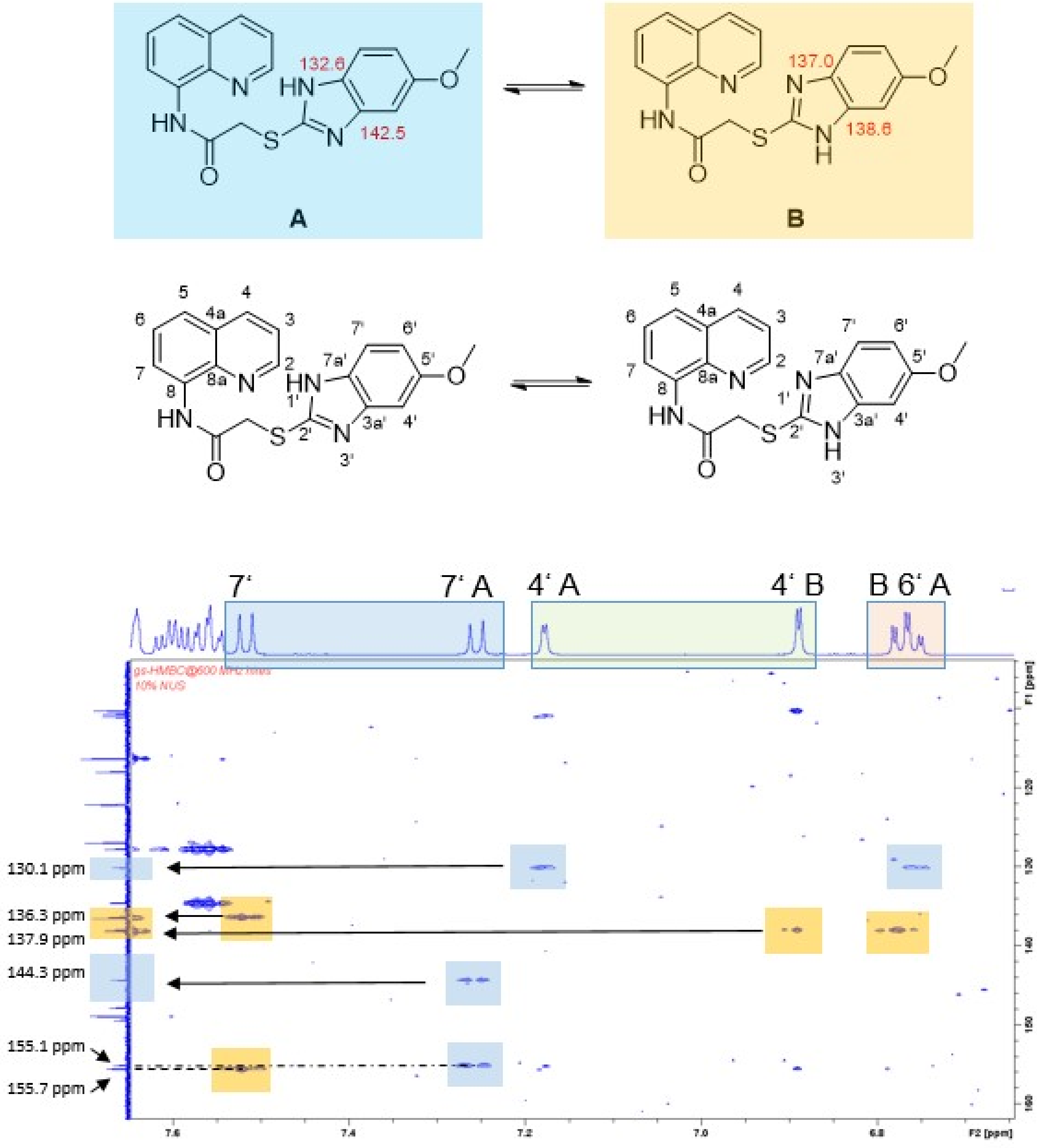

Figure S12: HMBC-spectra of a diluted **L1** sample proving the existence of two tautomeric structures of **L1**.

11. DFT Calculations

DFT calculations were performed with ORCA 5 of Neese and co-workers[2]. This DFT package was run on a MS Windows 10 Pro based (Version 21H1) PC system equipped with an AMD Ryzen Threadripper 3970X 32-Core and 128 GB RAM main memory in combination with the appropriate message passing interface MS-MPI 10.0.12498.5. MMFF optimized structures were used as starting geometries for the geometry optimizations with the recently published robust "Swiss army knife" composite method r^2SCAN-3c of Grimme and co-workers[3] with D4 dispersion correction and geometrical counter poise correction applying the modified triple-zeta basis set def2-mTZVPP. Subsequent frequency calculation of the final structure evidenced the absence of imaginary frequencies and thus the presence of true minima on the potential energy surface. In case of calculations that include a solvent, the Conductor-like Polarizable Continuum Model (CPCM) implemented in ORCA 5 was applied. For comparison, some calculations were performed with the common hybrid PBE0-D3 functional of Adamo and Barone[4] and the Karlsruhe triple-zeta basis set def2-TZVP by Weigend and Ahlrichs.[5]

DFT-calculated geometries and energies of tautomers A and B of the free ligands and corresponding zinc complex thereof (calculation method: r²SCAN-3c/def2-mTZVPP, DMSO via CPCM model)

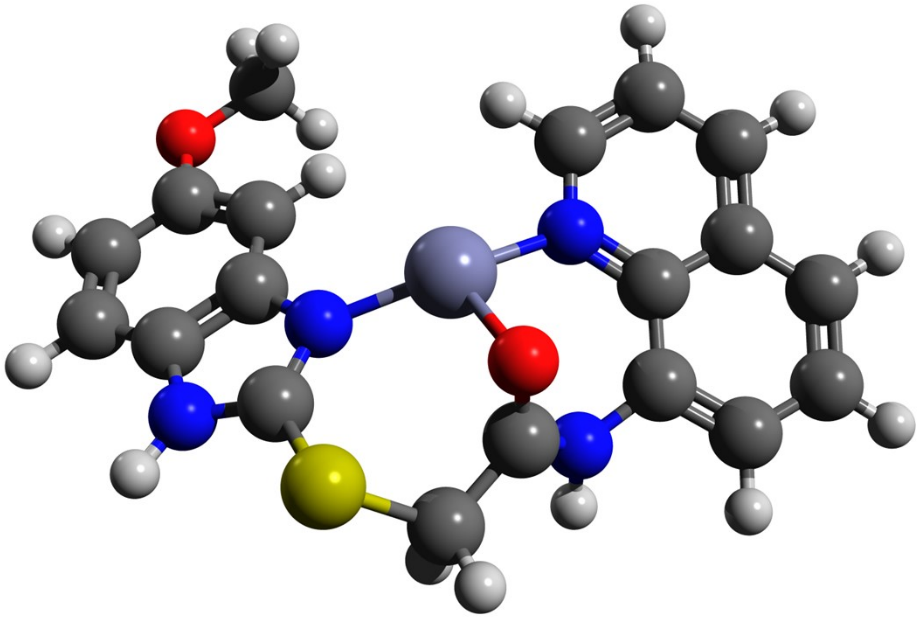

Fig. S13: DFT-calculated zinc complex **L1A-Zn** (tautomer A).

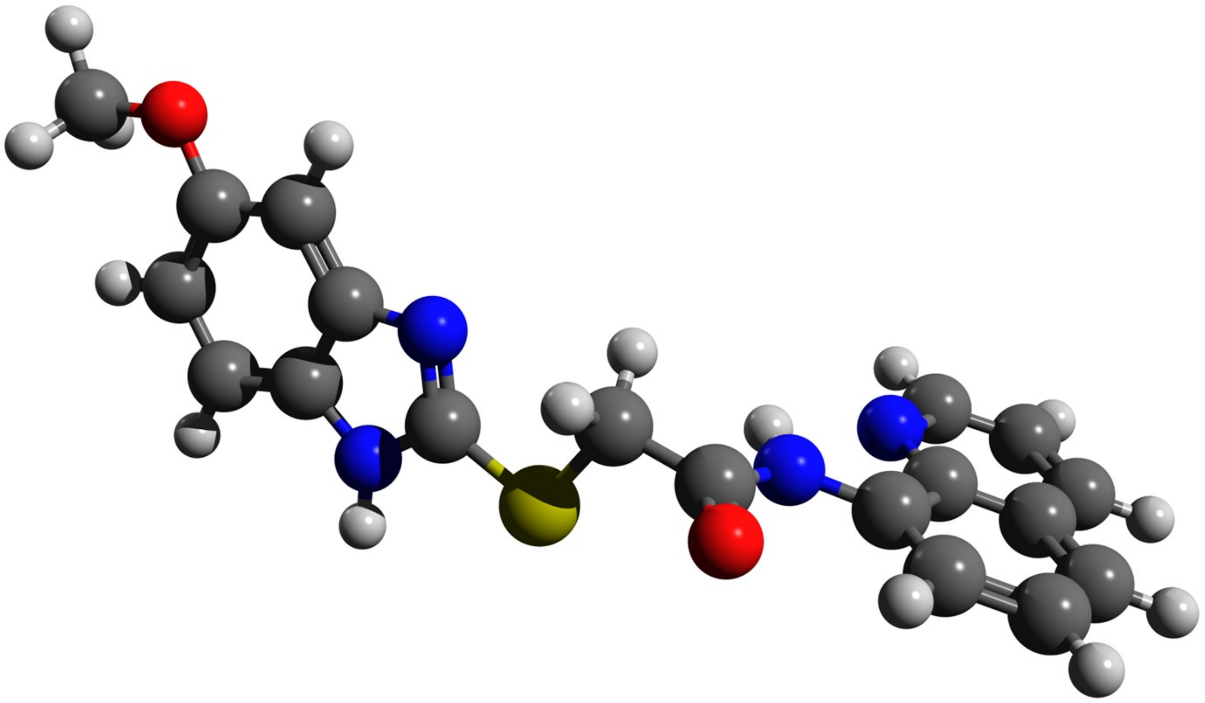

Fig. S14: DFT-calculated free ligand **L1A** (tautomer A).

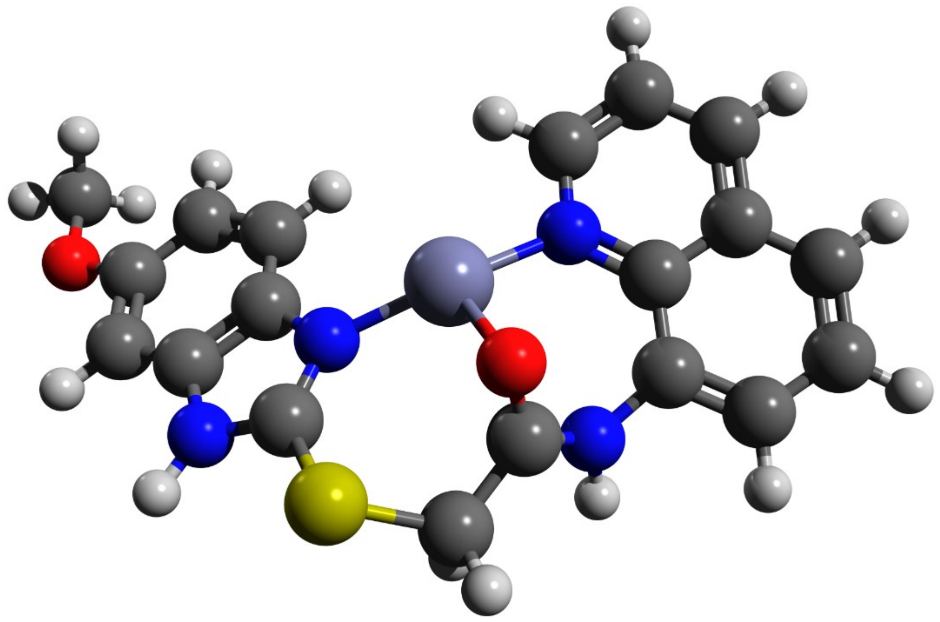

Fig. S15: DFT-calculated zinc complex **L1B-Zn** (tautomer B).

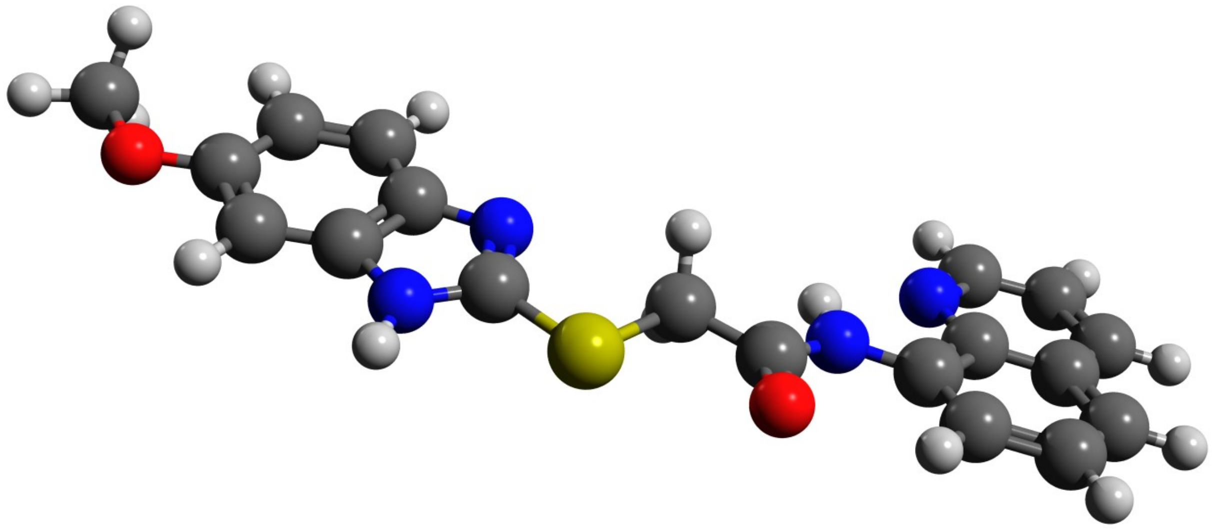

Fig. S16: DFT-calculated free ligand **L1B** (tautomer B).

DFT data of zinc complex **L1A-Zn** (tautomer A) in DMSO (refer to Fig. S13)

Energy: -3280.259377652987 ha   0 imaginary frequencies

```
---------------------------------
CARTESIAN COORDINATES (ANGSTROEM)
---------------------------------
  C      0.276801    3.406756    3.568595
  C      1.482275    3.773563    3.030957
  C      2.019051    3.034686    1.966990
  C      1.378676    1.926333    1.448997
  C      0.125952    1.511189    1.984989
  C     -0.420613    2.290853    3.055363
  N     -0.593034    0.439411    1.504189
  C     -1.800048    0.166244    2.007619
  C     -2.389888    0.900162    3.036897
  C     -1.688896    1.949574    3.570766
  N      2.020651    1.323314    0.319703
  C      2.425140    0.050924    0.244406
  C      3.294074   -0.319571   -0.934503
  O      2.044943   -0.807651    1.079707
  S      3.112858   -2.078081   -1.430815
  C      1.463929   -2.124915   -2.036946
  N      0.326229   -1.783484   -1.404909
  C     -0.719530   -2.093707   -2.272131
  C     -0.176944   -2.663475   -3.431460
  N      1.192139   -2.653068   -3.247314
  C     -2.094599   -1.919548   -2.111260
  C     -2.898704   -2.343903   -3.165786
  C     -2.344727   -2.923474   -4.333772
  C     -0.982923   -3.092737   -4.484011
  H     -0.172663    3.968386    4.382417
  H      2.024871    4.632391    3.411704
  H      2.967189    3.334548    1.529326
  H     -2.317032   -0.687372    1.574379
  H     -3.372601    0.618829    3.397330
  H     -2.098018    2.541077    4.385149
  H      4.345566   -0.234553   -0.627160
  H      3.131012    0.342397   -1.789883
  H      1.892743   -2.962086   -3.909632
  H     -2.492146   -1.476553   -1.204675
  H     -0.563809   -3.534012   -5.382190
  H      2.381575    1.972830   -0.371907
  Zn     0.137700   -0.971911    0.343278
  O     -4.252848   -2.245245   -3.174261
  C     -4.882542   -1.643593   -2.033783
  H     -4.686914   -2.226750   -1.125885
  H     -5.951046   -1.647713   -2.250648
  H     -4.536430   -0.612054   -1.895738
  H     -3.025819   -3.233511   -5.120041
```

DFT data of free ligand **L1A** (tautomer A) in DMSO (refer to Fig. S14)

Energy: -1501.119477374511 ha   0 imaginary frequencies

```
---------------------------------
CARTESIAN COORDINATES (ANGSTROEM)
---------------------------------
  C     -0.851438   -3.153010   -4.586745
  C     -0.495352   -2.063618   -3.830363
  C      0.603280   -2.101075   -2.946360
  C      1.355229   -3.254708   -2.821006
  C      1.010814   -4.410444   -3.595412
  C     -0.102606   -4.348713   -4.481769
  N      1.768970   -5.528880   -3.449839
  C      1.454122   -6.595245   -4.162535
  C      0.373182   -6.635839   -5.066607
  C     -0.401890   -5.513804   -5.223828
  N      2.461006   -3.410797   -1.980514
  H      2.865065   -4.342644   -2.026553
  C      3.030395   -2.504591   -1.140537
  C      4.182071   -3.090005   -0.337875
  O      2.662511   -1.339938   -1.009025
  H      4.852885   -3.680892   -0.968625
  S      5.100008   -1.699578    0.418785
  H      3.787412   -3.742447    0.449678
  C      6.374756   -2.633308    1.194085
  N      6.496119   -3.947555    1.216588
  C      7.648828   -4.178033    1.960359
  C      8.211342   -2.952354    2.381989
  N      7.368440   -1.977960    1.871981
  C      8.251268   -5.384309    2.304698
  C      9.417160   -5.332312    3.072378
  C      9.970598   -4.106484    3.487977
  C      9.367039   -2.896191    3.142742
  O      9.965293   -6.550485    3.380266
  C     11.155670   -6.565111    4.174412
  H     -1.698946   -3.114437   -5.265660
  H     -1.064938   -1.141719   -3.905093
  H      0.857746   -1.224854   -2.366590
  H      2.079456   -7.476967   -4.025114
  H      0.169413   -7.546093   -5.621592
  H     -1.244749   -5.503312   -5.910556
  H      7.467184   -0.978195    1.973849
  H      7.839403   -6.340203    1.995166
  H     10.875606   -4.083041    4.082771
  H      9.797916   -1.953306    3.465573
  H     11.413795   -7.617732    4.300281
  H     10.983094   -6.110714    5.158169
  H     11.978679   -6.045030    3.668373
```

DFT data of zinc complex **L1B-Zn** (tautomer B) in DMSO (refer to Fig. S15)

Energy: -3280.2579687025 ha 0 imaginary frequencies

```
---------------------------------
CARTESIAN COORDINATES (ANGSTROEM)
---------------------------------
  C      0.397364    3.321917    3.783992
  C      1.564445    3.742945    3.203120
  C      2.054151    3.075087    2.071599
  C      1.406830    1.981995    1.530588
  C      0.195002    1.510022    2.110795
  C     -0.307363    2.221137    3.248333
  N     -0.527969    0.448363    1.612028
  C     -1.707613    0.133500    2.155725
  C     -2.256632    0.803431    3.249140
  C     -1.542837    1.831457    3.807000
  N      1.987860    1.457645    0.331950
  C      2.416663    0.203225    0.160860
  C      3.191422   -0.076705   -1.107020
  O      2.127675   -0.707003    0.977823
  S      3.092622   -1.824881   -1.648429
  C      1.400114   -2.006662   -2.089061
  N      0.304005   -1.738666   -1.368942
  C     -0.785302   -2.179583   -2.122490
  C     -0.303968   -2.754450   -3.310548
  N      1.069254   -2.611952   -3.255547
  C     -2.149315   -2.149430   -1.859076
  C     -3.004712   -2.706017   -2.799246
  C     -2.505554   -3.282110   -3.989152
  C     -1.136579   -3.314286   -4.265173
  H     -0.017593    3.830365    4.649438
  H      2.110713    4.592157    3.599850
  H      2.968331    3.420534    1.596831
  H     -2.232931   -0.706011    1.705777
  H     -3.217213    0.488415    3.640054
  H     -1.917736    2.370339    4.672731
  H      4.259423    0.079700   -0.902080
  H      2.899590    0.596458   -1.918394
  H      1.732578   -2.884136   -3.969995
  H     -2.537622   -1.711696   -0.943317
  H     -0.763320   -3.758403   -5.181672
  H     -4.069641   -2.693976   -2.602549
  H      2.278009    2.149648   -0.351525
  Zn     0.182416   -0.912132    0.375655
  O     -3.299589   -3.840272   -4.939808
  C     -4.718170   -3.855739   -4.716319
  H     -4.967656   -4.426109   -3.814010
  H     -5.144850   -4.348103   -5.590660
  H     -5.115158   -2.837078   -4.636801
```

DFT data of free ligand L1B (tautomer B) in DMSO (refer to Fig. S16)

Energy: -1501.119901762734 ha   0 imaginary frequencies

```
---------------------------------
CARTESIAN COORDINATES (ANGSTROEM)
---------------------------------
  C     -0.741751   -3.219580   -4.819999
  C     -0.368463   -2.077775   -4.154543
  C      0.684860   -2.076363   -3.216315
  C      1.373858   -3.243735   -2.943175
  C      1.010400   -4.453434   -3.620111
  C     -0.056641   -4.430514   -4.563495
  N      1.706808   -5.583955   -3.330569
  C      1.374564   -6.700260   -3.953316
  C      0.335710   -6.782179   -4.902710
  C     -0.376950   -5.648593   -5.204964
  N      2.431108   -3.364277   -2.037093
  H      2.794032   -4.311907   -1.974524
  C      3.003586   -2.406238   -1.259273
  C      4.099999   -2.958705   -0.361429
  O      2.680552   -1.220988   -1.251458
  H      4.794988   -3.589856   -0.924011
  S      4.993701   -1.538671    0.366280
  H      3.656243   -3.564502    0.437163
  C      6.205367   -2.443084    1.268284
  N      6.315026   -3.749045    1.373080
  C      7.421107   -3.941023    2.200887
  C      7.966424   -2.695300    2.585754
  N      7.166560   -1.752228    1.972744
  C      8.008658   -5.114461    2.661776
  C      9.123899   -5.022535    3.493337
  C      9.647923   -3.768413    3.861163
  C      9.073594   -2.575669    3.409867
  H     -1.553842   -3.211007   -5.541863
  H     -0.888875   -1.143736   -4.346041
  H      0.954025   -1.159570   -2.710611
  H      1.950187   -7.590438   -3.701117
  H      0.114485   -7.732616   -5.377968
  H     -1.185892   -5.669000   -5.931060
  H      7.269352   -0.748752    2.021767
  H      7.610546   -6.086069    2.383215
  H      9.489944   -1.617035    3.702990
  O     10.738737   -3.611536    4.672475
  C     11.377254   -4.791881    5.171748
  H     12.208700   -4.440098    5.784309
  H     11.761898   -5.411678    4.352417
  H     10.691040   -5.383058    5.790670
  H      9.582778   -5.935359    3.853656
```

12. LOD-comparison of L1 to other Zn chemosensors (Table S1)

Chemosensor	LOD [µM]	Source
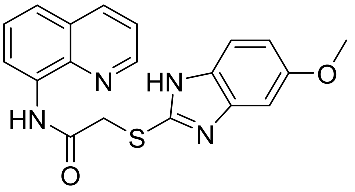	0.176	this work
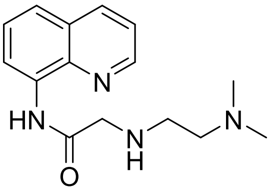	7.1	[6]
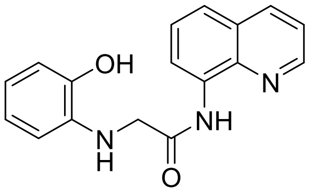	0.256	[7]
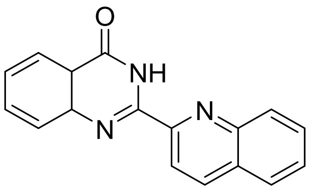	0.882	[8]
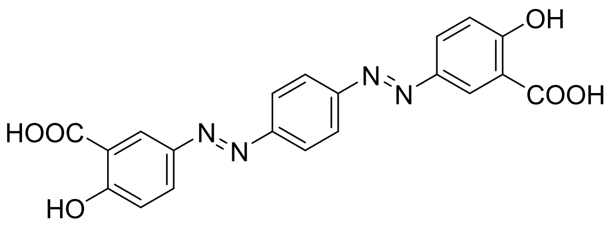	6.73	[9]
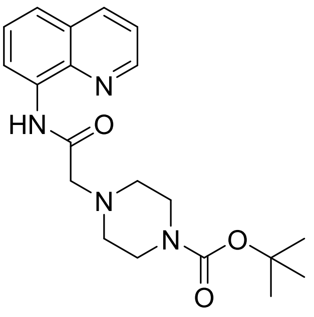	0.52	[10]

13. NMR-calculations of quaternary C atoms/ Δδ determination (Table S2)

	Tautomer A		Tautomer B	
**measured shift values**				
**C-N**		**144,3 (3a')**		**137,9 (7a')**
**C-NH**		**130,1 (7a')**		**136,3 (3a')**
	**Δδ**	**14,2**	**Δδ**	**1,6**
**Spartan´20 (ωB97X-D/6-31G*)**				
**C-N**		**144,3**		**138,8**
**C-NH**		**128,5**		**136,1**
	**Δδ**	**15,8**	**Δδ**	**2,7**
ACD2018 (2018.2.5)				
**C-N**		142,5		137,0
**C-NH**		132,6		138,6
	Δδ	9,9	Δδ	-1,6

14. Proposed binding-mode

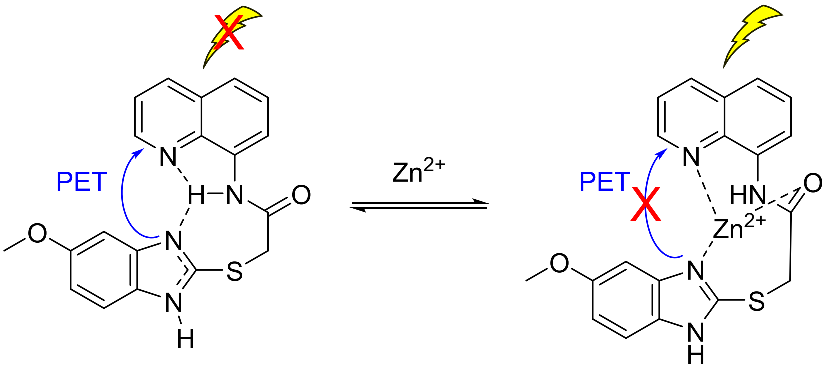

Fig. S17: Proposed binding-mode of **L1**.

15. Sources/References

[1] a) A. G. Mwalupindi, A. Rideau, R. A. Agabaria, I. M. Warner, *Talanta* 1994, *41*, 599; b) G. Wang, J. Qin, L. Fan, C.-R. Li, Z. Yang, *J. Photochem. Photobiol. A* 2016, *314*, 29.

[2] (a) F. Neese, The ORCA Program System, *WIREs Comput. Mol. Sci.* **2012**, 2: 73-78; doi: 10.1002/wcms.81);

b) F. Neese, Software Update: The ORCA Program System, Version 4.0. *Wiley Interdiscip. Rev. Comput. Mol. Sci.* **2018**, *8*, e1327, doi: 10.1002/wcms.1327.

[3] S. Grimme, A. Hansen, S. Ehlert, and J.-M. Mewes, r²SCAN-3c: A "Swiss army knife" composite electronic-structure method, *J. Chem. Phys.* **2021**, *154*, 064103; https://doi.org/10.1063/5.0040021.

[4] C. Adamo, V. Barone, Toward Reliable Density Functional Methods Without Adjustable Parameters: The PBE0 Model, *J. Chem. Phys.* **1999**, *110*, 6158–6170; https://doi: 10.1063/1.478522.

[5] F. Weigend, R. Ahlrichs, Balanced basis sets of split valence, triple zeta valence and quadruple zeta valence quality for H to Rn: Design and assessment of accuracy. *Phys. Chem. Chem. Phys.* **2005**, *7*, 3297–3305; doi.org/10.1039/b508541a.

[6] H. Kim, J. Kang, K. B. Kim, E. J. Song, C. Kim, *Spectrochim. Acta A* **2014**, *118*, 883.

[7] Y. Yue, Q. Dong, Y. Zhang, Y. Sun, Y. Gong, *Anal. Methods* **2015**, *7*, 5661.

[8] X.-J. Bai, J. Ren, J. Zhou, Z.-B. Song, *Heterocycl. Commun.* **2018**, *24 (3)*, 135.

[9] M. Mariyappan, N. Malini, J. Sivamani, G. Sivaraman, M. Harikrishnan, S. Murugesan, A. Siva, *J. Fluoresc.* **2019**, *29*, 737.

[10] N.-N. Li, Y.-Q. Ma, S. Zeng, Y.-T. Liue, X.-J. Sun, Z.-Y. Xing, *Synth. Met.* **2017**, *232*, 17.
